# Multivariate assessment of newly developed guava (*Psidium guajava* L.) hybrids for tree and fruit quality traits

**DOI:** 10.1038/s41598-026-45320-8

**Published:** 2026-04-04

**Authors:** Ankita Kashyap, Prabhanshu Mishra, Chavlesh Kumar, Amit Kumar Goswami, Jai Prakash, Rakesh Singh, Shalini Gaur Rudra, Shailendra Kumar Jha, Narendra Singh, Rajeev Ranjan Kumar, Kritidipta Pramanik, M. K. Sushravya, Paresh Chaukhande, R. M. Sharma, Sanjay Kumar Singh

**Affiliations:** 1 Division of Fruits and Horticultural Technology, ICAR-Indian Agricultural Research Institute, New Delhi, 110012 India; 2 Division of Genomic Resources, ICAR- National Bureau of Plant Genetic Resources, New Delhi, 110012 India; 3 Division of Food Science and Post-Harvest Technology, ICAR-Indian Agricultural Research Institute, New Delhi, 110012 India; 4 Division of Genetics, ICAR-Indian Agricultural Research Institute, New Delhi, 110012 India; 5 School of Crop Science, ICAR-Indian Agricultural Research Institute, Hazaribag, Jharkhand 825405 India; 6 Division of Forecasting & Agricultural Systems Modelling, ICAR-Indian Agricultural Statistics Research Institute, New Delhi, 110012 India; 7 Division of Vegetable Science, ICAR-Indian Agricultural Research Institute, New Delhi, 110012 India; 8 Horticultural Science Division, ICAR-Krishi Anusandhan Bhawan-II, New Delhi, 110012 India

**Keywords:** Full-sib, Morphological, Biochemical, Nutritional value, MGIDI, Biotechnology, Genetics, Plant sciences

## Abstract

Guava (*Psidium guajava* L.) is an important commercial fruit crop valued for its exceptional nutraceutical value and pharmacological properties. Growing awareness about guava’s health benefits is driving demand for higher-quality, nutraceutical-rich cultivars. Varietal development and genetic improvement in guava involve crossing between heterozygous elite parents to generate segregating full-sib populations for future selection. In this study, hybridization between Allahabad Safeda and Arka Kiran, was attempted, and hybrid progenies were raised. A total of 50 hybrid progenies were confirmed as hybrid with three SSRs (FHTGSSR-3.4, FHTGSSR-7.5, and FHTGSSR-3.6). These confirmed hybrid progenies exhibited wide phenotypic variability with both intermediate and transgressive segregation across tree, leaf, fruit physical and biochemical parameters. Fruit parameters varied markedly in size (length: 5.59–10.13 cm; weight: 79.33–406.33 g) while qualitative variation was evident in leaf shape, fruit shape, peel, and pulp colour. Biochemical profiling revealed high variability in respect of TSS (10.30–14.80°Brix), ascorbic acid (65.00–302.00 mg/100 g), total carotenoids (0.78–9.55 mg/100 g), lycopene (0.53–9.03 mg/100 g) and antioxidant activities (DPPH: 86–95%; FRAP: 8.9–22.0 µmol Trolox/g). MGIDI analysis identified seven guava hybrids as superior multi-trait performers amongst the studied guava hybrids. Multivariate analyses helped characterize the underlying trait variability, and hierarchical clustering grouped progenies into distinct clusters. Additionally, transgressive segregation for key nutraceutical traits underscores the potential for selecting superior recombinants. Overall, this study highlights the effectiveness of hybridization between heterozygous guava genotypes in generating genetic variability, while establishing SSR markers are robust and reliable tools for confirming hybridity in crop improvement programs.

**Supplementary Information:**

The online version contains supplementary material available at 10.1038/s41598-026-45320-8.

## Introduction

Guava (*Psidium guajava* L.) is one of the important commercially cultivated fruit crops throughout the tropical and subtropical regions of the world. The guava is recognized for its high nutraceutical potential and wider adaptability across edaphic and ecological conditions. The fruit is nutritionally rich, with higher levels of vitamin C, vitamin A, and vitamin B-complex, as well as a fair source of essential mineral nutrients (iron, calcium, zinc, and potassium), dietary fiber, and antioxidants^1–3^. Several studies have established the nutraceutical properties and health benefits of guava^4,5^. Owing to its rich nutritional profile, large availability and popularity, it is often referred to as the “Apple of the Tropics” and “Super fruit”^6^. Guava has emerged as a highly remunerative fruit crop, offering substantial economic benefits to growers and serving as an essential source of nutrition, ensuring nutritional security.

The guava is native to tropical America^7^; however, it has been well established and widely cultivated across different continents, with major production in Asia. India has currently emerged as the world’s largest producer of guava, recording an annual production of 5.37 million metric tons from 358 thousand hectares^8^. While over 400 cultivars exist globally, only a few, such as ‘Allahabad Safeda’, ‘Sardar’, ‘Lalit’, ‘Shweta’, ‘Arka Mridula’, and hybrids like ‘Arka Amulya’ ‘Hisar Safeda’, and ‘Arka Kiran’ are commercially cultivated in India^9,10^. Despite their commercial relevance, most guava cultivars lack critical horticultural traits, such as a compact growth habit, thick pulp with uniform size, low number and soft seeds, high lycopene content, prolonged shelf-life, and resistance to biotic and abiotic stresses^9,11,12^. Therefore, it is imperative to tailor guava cultivars with superior fruit quality and enhanced nutraceutical properties to meet the growing demands of the industry. Being a perennial fruit crop, guava breeding is constrained by high heterozygosity, long juvenile phase with a lengthy breeding cycle, resource-intensive field evaluations and clonal trials that last several years, and is further complicated by limited knowledge of the inheritance patterns of key traits. Despite various challenges, hybridization between two desirable heterozygous parents is routinely employed in perennial fruit crops, including guava, to generate segregating populations and identify superior recombinants as improved genotypes. Moreover, these crops provide unique opportunities to fix heterosis and recombination through vegetative propagation. Therefore, a systematic guava genetic improvement program was initiated to improve fruit quality and enhance nutraceutical attributes with desirable organoleptic qualities^13,14^.

Integrating targeted morpho-biochemical and molecular approaches is essential in the breeding programs of perennial fruit crops, including guava^15^. Morphological parameters have traditionally been employed to characterize plant genotypes and assess genetic relationships^16^, which utilize a defined set of morphological traits from descriptors to classify genotypes and capture phenotypic diversity^17,18^. Biochemical characterization, which involves the identification and quantification of genotype-specific metabolites, provides insights into nutritional and functional properties and aids in the discovery of biochemical markers linked to fruit quality and nutritional traits^19^. The use of molecular markers, particularly simple sequence repeat (SSR) markers, enhances precision in identifying hybrids, assessing genetic diversity, and detecting polymorphisms among progenies^20^. SSRs are co-dominant, reproducible, and highly informative, making them ideal for guava genetic studies, including hybrid confirmation, genetic distinctiveness, fingerprinting, and diversity analysis. Furthermore, segregating populations derived from biparental crosses also offer a powerful platform for dissecting the inheritance of key morphological and biochemical traits, unravelling genotype–phenotype relationships, and accelerating the genetic improvement of guava cultivars.

This study aimed to develop full-sib guava progenies by crossing Allahabad Safeda (♀) with Arka Kiran (♂) and to identify desirable novel hybrids. The newly developed hybrid progenies, along with parental genotypes, were evaluated for morphological and biochemical parameters, in addition to SSR-based hybridity confirmation. Furthermore, to understand the extent of morpho-biochemical variation among the hybrids, multivariate analyses, including principal component analysis (PCA), hierarchical clustering and correlation were performed. This combined evaluation approach enables the identification of superior guava hybrids with desirable fruit parameters, thereby supporting future breeding programmes and facilitating the commercial exploitation of elite hybrids.

## Results

In this study, morpho-biochemical parameters of 50 novel hybrids, along with parental genotypes (Allahabad Safeda and Arka Kiran), were evaluated, and the results are enumerated hereunder.

### SSR-based hybridity confirmation

**Fig. 1:**
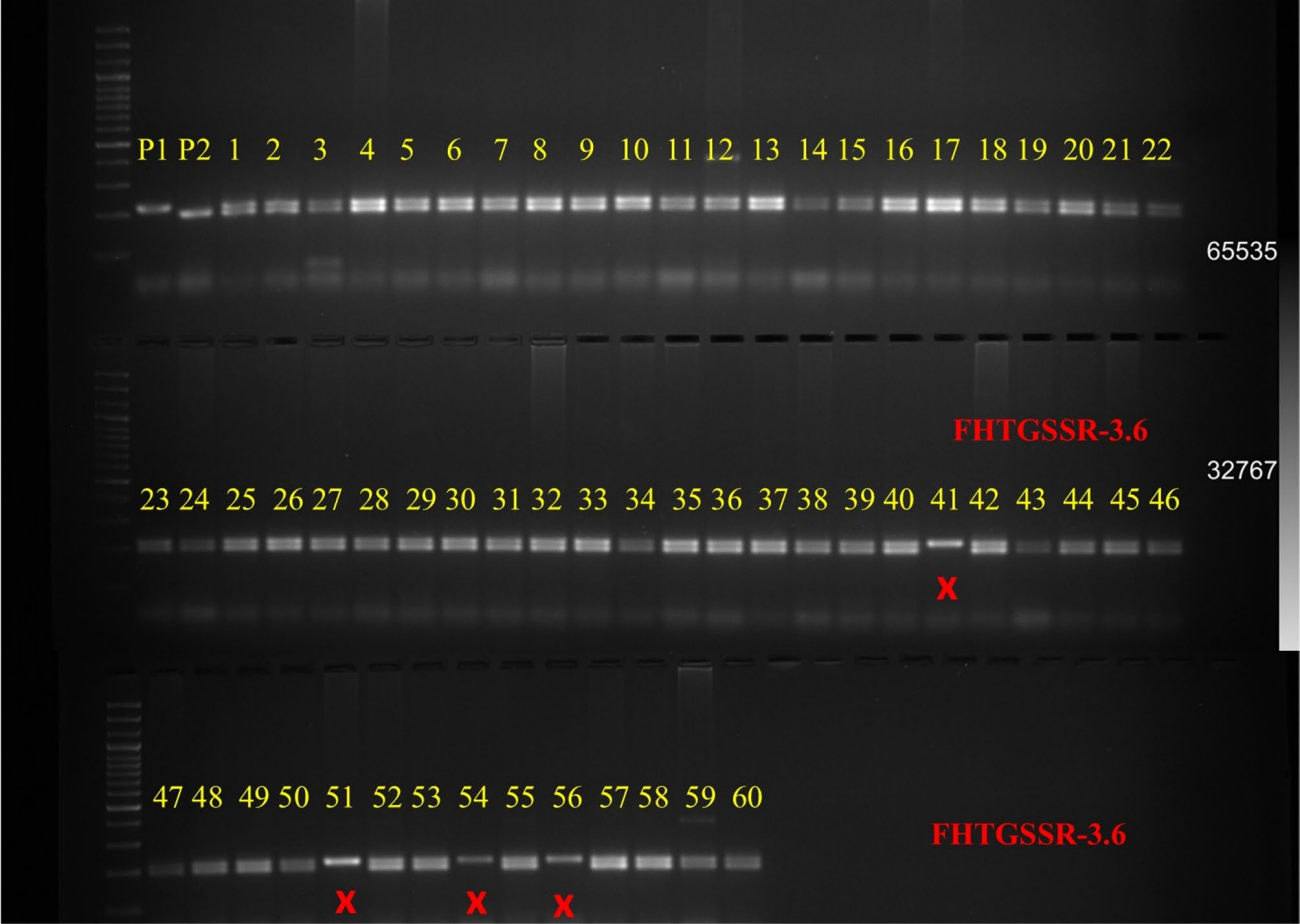
Gel image of SSR locus, FHTGSSR-3.6 ascertaining the hybridity among the full-sib progenies of Allahabad Safeda and Arka Kiran. P_1_-Allahabad Safeda, P_2_-Arka Kiran, while numbers 1–60 denote the guava progenies evaluated for hybridity. Cross symbols (×) indicate progenies not validated as hybrids.

A total of 46 g-SSR markers distributed across the guava genome were initially screened to identify polymorphic homozygous alleles between the parental genotypes ‘Allahabad Safeda’ (female) and ‘Arka Kiran’ (male). Among these, three markers FHTGSSR-3.4, FHTGSSR-7.5, and FHTGSSR-3.6 were found highly informative, exhibiting clear, reproducible homozygous polymorphic alleles between the parents (Fig. 1). The selected markers consistently amplified distinct allele sizes specific to each parent, enabling unambiguous molecular discrimination. When applied to full-sib progeny, these markers successfully validated hybridity by detecting the presence of both parental alleles in the hybrid progenies, thereby confirming the inheritance of allelic segments from each parent. Out of the 60 progenies analyzed, 56 genotypes exhibited both female and male-specific alleles, thereby providing robust molecular evidence of their hybrid status. Of the 56 SSR-confirmed hybrid progenies, 50 attained uniform bearing and were subsequently selected for multivariate analyses.

### Morphological characterization

**Fig. 2:**
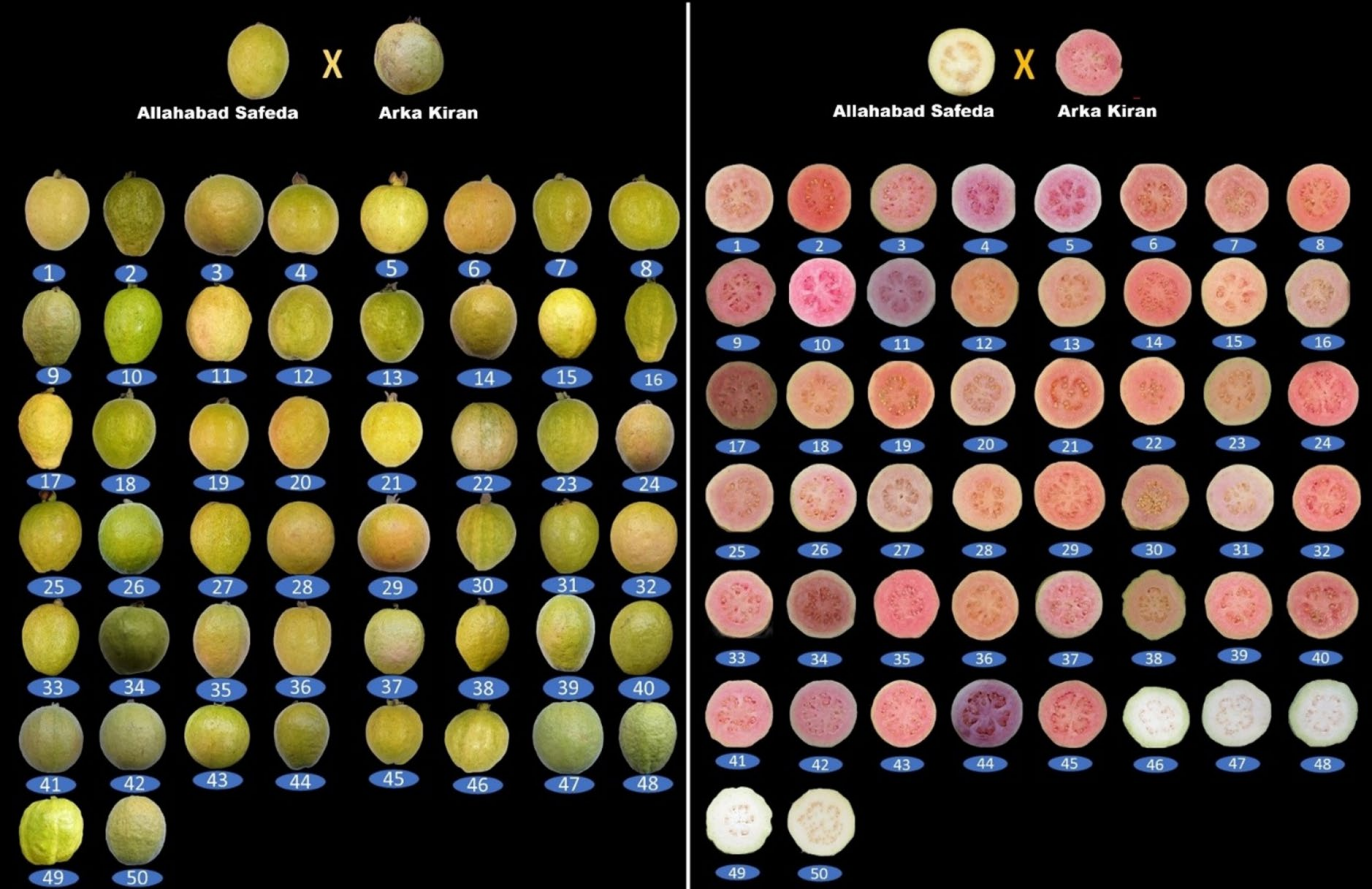
The newly developed guava hybrid progenies and parental genotypes evaluated in present study. P_1_-Allahabad Safeda, P_2-_Arka Kiran, 1. GH20_1A, 2. GH20_1B, 3. GH20_1C, 4. GH20_1D, 5. GH20_10D, 6. GH20_2B, 7. GH20_2C, 8. GH20_2E, 9. GH20_3A, 10. GH20_3B, 11. GH20_3C, 12. GH20_3D, 13. GH20_4B, 14. GH20_5A, 15. GH20_5C, 16. GH20_5D, 17. GH20_5E, 18. GH20_6A, 19. GH20_6B, 20. GH20_6D, 21. GH20_6E, 22. GH20_7C, 23. GH20_7E, 24. GH20_8A, 25. GH20_8B, 26. GH20_8C, 27. GH20_8D, 28. GH20_8E, 29. GH20_9A, 30. GH20_9B, 31. GH20_10A, 32. GH20_11B, 33. GH20_11C, 34. GH20_11D, 35. GH20_12B, 36. GH20_12E, 37. GH20_13B, 38. GH20_13E, 39. GH20_14D, 40. GH20_15B, 41. GH20_15E , 42. GH20_18B, 43. GH20_16D,44. GH20_17B, 45. GH20_17E, 46. GH20_5B, 47. GH20_11A, 48. GH20_12C, 49. GH20_18A, 50. GH20_20B.

A wide range of morphological variation was observed among the 50 studied guava hybrid progenies along with their two parental genotypes (Table 1; Supplementary Table 1 & 2; Fig. 2). The tree branching habit was noted as drooping, spreading, and erect among the studied hybrid progenies and parental genotypes, 21 hybrids along with both parents, showed spreading canopy habit, whereas 18 exhibited an erect habit, while 11 showed drooping branches. The colour of young shoots also showed significant variations, where 28 hybrids had green shoots with red streaks, while 18 progenies and both the parent genotypes showed entirely green young shoots. Interestingly, six hybrids exhibited dark red-coloured young shoots among the studied hybrid progenies.

**Table 1:** Frequency distribution of qualitative morphological parameters among the newly developed guava hybrid progenies and parental genotypes.

Parameters	1	2	3	4	5
Tree: attitude of branches	Erect (18)	Spreading (23)	Drooping (11)		
Young shoot: colour of stem	Green (18)	Green with red streaks (28)	Dark Red (6)		
Leaf: shape	Ovate (21)	Obovate (11)	Oblong (11)	Lanceolate (1)	Oblanceolate (8)
Leaf: shape of tip	Acute (12)	Obtuse (29)	Rounded (11)		
Leaf: shape of base	Obtuse (7)	Rounded (29)	Cordate (16)		
Petiole orientation	Twisted (28)	Straight (24)			
Leaf: twisting	Absent (15)	Present (37)			
Young leaf: anthocyanin colouration	Absent (34)	Present (18)			
Leaf: variegation	Absent (52)	Present (0)			
Leaf: colour	Green group (52)				
Leaf: pubescence on lower side	Sparse (52)	Dense (0)			
Colour of lamina (upper and lower surface)	Green (25)	Light green (10)	Dark green (17)		
Lamina thickness	Thin (14)	Intermediate (21)	Thick (17)		
Leaf lamina pubescence	Absent (14)	Sparse (15)	Medium (2)	Dense (15)	Very dense (6)
Colour of leaf during winter	Pink (1)	Red (14)	Coppery (22)	Brick Red (10)	Brown (5)
Fruit Shape at stalk end	Broadly rounded (5)	Rounded (31)	Pointed (13)	Necked (3)	
Fruit prominence of neck	Absent (44)	Present (8)			
Fruit relief of surface	Smooth (32)	Rough (20)			
Fruit longitudinal ridges	Absent (33)	Present (19)			
Fruit longitudinal grooves	Absent (40)	Present (12)			
Fruit puffiness	Absent (50)	Present (2)			
Fruit ridged collar around calyx cavity	Inconspicuous (52)	Conspicuous (0)			
Fruit: Pulp colour	Red group (46)	White group (6)			
Fruit: Peel colour	Yellow group (31)	Green yellow group (15)	Yellow green (4)	Yellow orange (2)	

The parental guava genotype, Allahabad Safeda, had obovate leaves, whereas Arka Kiran exhibited oblanceolate leaves (Supplementary Fig. 1). Among hybrid progenies, 21 had ovate leaf shape, 10 had obovate, 11 had oblong, 7 had oblanceolate, while 1 hybrid exhibited lanceolate leaf shape. Significant variation was also noted for leaf tip, i.e., 28 progenies showed obtuse tips, which were most common, each of 11 progenies displayed rounded and acute leaf tips, while Allahabad Safeda had obtuse and Arka Kiran had acute leaf tip. The leaf base was observed as round in 28 progenies, 15 had cordate, while it was noted as obtuse in 7 progenies; parental genotypes Allahabad Safeda had cordate, and Arka Kiran had a rounded leaf base. The twisted leaf was observed in 37 hybrids, but the same attribute was absent in 15 hybrids, including both parents. Anthocyanin pigmentation on leaves was observed in Arka Kiran and 17 hybrids, but it was absent in Allahabad Safeda and another 33 hybrid progenies. All hybrids displayed green leaf colour on the adaxial surface, with sparse pubescence on the abaxial side. The intermediate thickness of the leaf lamina was most common, observed in 19 progenies and both parents. Pubescence density on leaf surfaces ranged from very dense to absent, indicating broad variability among progenies. Distinct variability was also recorded in leaf colour development during winter amongst the hybrid guava progenies and parents. Allahabad Safeda developed a coppery hue, while Arka Kiran exhibited red colour. Among the progenies, coppery colour was most common (21 hybrids), followed by red (13 hybrids), brick red (10 hybrids), and brown (5 hybrids) leaf colour. However, one genotype, GH20_18B, displayed a unique pink winter leaf colour.

Furthermore, marked variability was observed among hybrid progenies and parental genotypes for leaf length, leaf width, and petiole length, indicating considerable variation and genetic divergence in leaf-related parameters (Table 2). The leaf length ranged from 11.23 to 15.9 cm, with the longest leaf in the hybrid GH20_8A and the shortest in the hybrid GH20_5C. The widest leaf blade (7.37 cm) was recorded in GH20_13B, while GH20_13E exhibited the narrowest leaf blade (4.80 cm). The ratio of leaf length to leaf width, which indicates the overall leaf shape index, varied from 1.92 to 2.70. The guava hybrid, GH20_18B, had the highest ratio (2.70), reflecting an elongated leaf lamina, whereas GH20_8E showed the lowest ratio (1.92), suggesting a comparatively broader lamina. The petiole length varied from 0.50 to 0.83 cm, and the guava hybrid GH20_14D has the shortest petiole, while the longest petioles occurred in hybrid GH20_11A.

**Table 2:** Variations in leaf parameters among the newly developed guava hybrid progenies and parental genotypes.

S. No	Guava genotype	Leaf length (cm)	Leaf width (cm)	Leaf length/width	Petiole length (cm)
1	Allahabad Safeda (P_1_)	11.53 ± 0.29^st^	5.73 ± 0.18^l–r^	2.02 ± 0.07^n–p^	0.6 ± 0.06^c–f^
2	Arka Kiran (P_2_)	13.87 ± 0.24^f–k^	5.87 ± 0.07^j–o^	2.36 ± 0.04^f–h^	0.5 ± 0.06^ef^
3	GH20_1A	14.93 ± 0.23^b–d^	6.83 ± 0.18^b–e^	2.19 ± 0.08^h–l^	0.7 ± 0.06^a–d^
4	GH20_1B	14.43 ± 0.18^d–f^	5.43 ± 0.18^n–t^	2.66 ± 0.05^ab^	0.57 ± 0.03^c–f^
5	GH20_1C	12.17 ± 0.23^q–s^	5.33 ± 0.22^q–t^	2.3 ± 0.07^f–i^	0.67 ± 0.09^a–e^
6	GH20_1D	12.57 ± 0.24^pq^	5.43 ± 0.27^n–t^	2.32 ± 0.08^f–h^	0.6 ± 0.06^c–f^
7	GH20_10D	14.47 ± 0.15^c–f^	6.53 ± 0.09^c–g^	2.23 ± 0.03^g–k^	0.67 ± 0.09^a–e^
8	GH20_2B	13.53 ± 0.47^i–m^	5.63 ± 0.09^m–s^	2.4 ± 0.06^d–f^	0.5 ± 0.06^ef^
9	GH20_2C	13.2 ± 0.23^l–p^	5.50 ± 0.21^n–t^	2.4 ± 0.05^d–f^	0.57 ± 0.09^c–f^
10	GH20_2E	12.43 ± 0.12^q^	5.40 ± 0.12^o–t^	2.3 ± 0.03^f–i^	0.70 ± 0.06^a–d^
11	GH20_3A	13.4 ± 0.12^j–m^	5.27 ± 0.12^r–u^	2.55 ± 0.04^a–c^	0.80 ± 0.06^ab^
12	GH20_3B	13.53 ± 0.22^i–m^	6.33 ± 0.12^f–j^	2.14 ± 0.01^k–n^	0.57 ± 0.03^c–f^
13	GH20_3C	13.1 ± 0.27^m–p^	6.00 ± 0.06^h–m^	2.18 ± 0.03^h–l^	0.63 ± 0.03^b–e^
14	GH20_3D	11.3 ± 0.25^t^	5.37 ± 0.19^p–t^	2.11 ± 0.03^j–n^	0.43 ± 0.03^f^
15	GH20_4B	13.1 ± 0.27^m–p^	6.37 ± 0.15^e–i^	2.06 ± 0.06^l–o^	0.60 ± 0.06^c–f^
16	GH20_5A	14.4 ± 0.15^d–g^	6.17 ± 0.07^g–l^	2.34 ± 0.02^f–h^	0.70 ± 0.06^a–d^
17	GH20_5C	11.23 ± 0.18^t^	5.23 ± 0.09^s–u^	2.15 ± 0.01^j–n^	0.70 ± 0.06^a–d^
18	GH20_5D	14.23 ± 0.15^e–h^	6.23 ± 0.15^g–k^	2.28 ± 0.03^f–j^	0.73 ± 0.07^a–c^
19	GH20_5E	13.7 ± 0.27^h–m^	5.87 ± 0.12^j–o^	2.34 ± 0.03^f–h^	0.50 ± 0.06^ef^
20	GH20_6A	12.2 ± 0.23^qr^	5.53 ± 0.15^m–t^	2.21 ± 0.02^h–l^	0.70 ± 0.06^a–d^
21	GH20_6B	12.13 ± 0.35^q–s^	5.5 ± 0.23^n–t^	2.21 ± 0.03^g–k^	0.53 ± 0.09^d–f^
22	GH20_6D	13.37 ± 0.27^j–m^	5.57 ± 0.27^m–t^	2.41 ± 0.07^d–f^	0.60 ± 0.06^c–f^
23	GH20_6E	11.33 ± 0.22^t^	5.30 ± 0.15^r–t^	2.14 ± 0.04^j–n^	0.70 ± 0.06^a–d^
24	GH20_7C	15.5 ± 0.17^ab^	7.10 ± 0.15^ab^	2.18 ± 0.05^h–l^	0.60 ± 0.06^c–f^
25	GH20_7E	11.63 ± 0.12^r–t^	5.57 ± 0.2^m–t^	2.09 ± 0.06^k–n^	0.60 ± 0.06^c–f^
26	GH20_8A	15.97 ± 0.39^a^	7.07 ± 0.24^ab^	2.26 ± 0.02^f–j^	0.67 ± 0.03^a–e^
27	GH20_8B	12.1 ± 0.12^q–s^	5.57 ± 0.22^m–t^	2.18 ± 0.07^i–m^	0.5 ± 0.06^ef^
28	GH20_8C	14.63 ± 0.18^c–e^	5.83 ± 0.12^k–p^	2.51 ± 0.02^b–d^	0.73 ± 0.03^a–c^
29	GH20_8D	13.27 ± 0.18^k–n^	6.40 ± 0.15^d–h^	2.07 ± 0.03^l–o^	0.6 ± 0.06^c–f^
30	GH20_8E	13.23 ± 0.15^k–o^	6.90 ± 0.27^a–c^	1.92 ± 0.05^p^	0.73 ± 0.03^a–c^
31	GH20_9A	15.37 ± 0.19^ab^	7.13 ± 0.09^ab^	2.15 ± 0.02^j–n^	0.60 ± 0.06^c–f^
32	GH20_9B	12.70 ± 0.17^n–q^	6.17 ± 0.18^g–l^	2.06 ± 0.03^l–o^	0.70 ± 0.06^a–d^
33	GH20_10A	12.60 ± 0.06^o–q^	5.9 ± 0.27^i–n^	2.14 ± 0.09^i–m^	0.70 ± 0.06^a–d^
34	GH20_11B	14.17 ± 0.2^e–h^	6.13 ± 0.20^g–l^	2.31 ± 0.04^f–i^	0.73 ± 0.03^a–c^
35	GH20_11C	14.17 ± 0.2^e–h^	5.90 ± 0.17^i–n^	2.40 ± 0.04^d–f^	0.70 ± 0.06^a–d^
36	GH20_11D	13.63 ± 0.32^h–m^	6.50 ± 0.23^c–g^	2.10 ± 0.03^k–n^	0.60 ± 0.06^c–f^
37	GH20_12B	13.57 ± 0.18^i–m^	5.80 ± 0.12^k–q^	2.34 ± 0.05^e–g^	0.57 ± 0.03^c–f^
38	GH20_12E	12.37 ± 0.19^q^	5.23 ± 0.09^s–u^	2.36 ± 0.02^e–g^	0.67 ± 0.09^a–e^
39	GH20_13B	14.43 ± 0.12^d–f^	7.37 ± 0.09^a^	1.96 ± 0.03^op^	0.63 ± 0.09^b–e^
40	GH20_13E	12.20 ± 0.21^q–r^	4.80 ± 0.10^u^	2.55 ± 0.09^b–d^	0.60 ± 0.06^c–f^
41	GH20_14D	14.50 ± 0.06^c–f^	6.40 ± 0.12^d–h^	2.27 ± 0.04^f–j^	0.50 ± 0.06^ef^
42	GH20_15B	14.00 ± 0.56^e–j^	5.90 ± 0.06^i–n^	2.37 ± 0.07^e–g^	0.60 ± 0.12^c–f^
43	GH20_15E	11.43 ± 0.12^t^	5.13 ± 0.09^tu^	2.23 ± 0.04^g–k^	0.60 ± 0.06^c–f^
44	GH20_18B	14.47 ± 0.2^c–f^	5.37 ± 0.13^p–t^	2.70 ± 0.04^a^	0.60 ± 0.06^c–f^
45	GH20_16D	14.47 ± 0.09^c–f^	6.23 ± 0.24^g–k^	2.32 ± 0.08^f–h^	0.70 ± 0.06^a–d^
46	GH20_17B	14.43 ± 0.12^d–f^	6.83 ± 0.09^b–e^	2.11 ± 0.01^k–n^	0.50 ± 0.06^ef^
47	GH20_17E	11.27 ± 0.15^t^	5.50 ± 0.15^n–t^	2.05 ± 0.04^l–o^	0.50 ± 0.06^ef^
48	GH20_5B	15.10 ± 0.27^bc^	6.77 ± 0.27^b–f^	2.05 ± 0.13^m–p^	0.80 ± 0.06^ab^
49	GH20_11A	13.67 ± 0.29^h–m^	6.13 ± 0.18^g–l^	2.47 ± 0.11^c–e^	0.83 ± 0.03^a^
50	GH20_12C	15.10 ± 0.23^bc^	6.87 ± 0.24^b–d^	2.08 ± 0.03^l–o^	0.80 ± 0.06^ab^
51	GH20_18A	11.53 ± 0.29^st^	6.53 ± 0.15^c–g^	2.09 ± 0.00^k–n^	0.80 ± 0.06^ab^
52	GH20_20B	13.87 ± 0.24^f–k^	6.73 ± 0.22^b–f^	2.24 ± 0.04^g–k^	0.70 ± 0.06^a–d^
	Range	11.2–16.0	4.8–7.4	1.9–2.7	0.43–0.83
	Mean	13.42	6.0	2.25	0.64
	LSD (*p* ≤ 0.05)	0.66	0.47	0.15	0.17
	CV (%)	9.15	10.34	7.60	15.32

Values are mean ± SE. Means followed by different superscript letters within a column represent sampling significant differences.

### Fruit physical parameters

The 50 hybrid progenies along with parental genotypes, Arka Kiran and Allahabad Safeda, showed significant variation in fruit physical parameters (Table 3 & Fig. 2). Fruit shape at the stalk end was observed as rounded, pointed, broadly rounded, and necked among the hybrids and parental genotypes. Arka Kiran and most of the hybrid progenies (30 Nos.) exhibited a rounded shape, while Allahabad Safeda, along with 12 progenies, showed a pointed form. Additionally, 5 progenies were broadly rounded, and 3 were necked (Supplementary Table 2). The fruit peel colour was noted as Yellow Group (3C) in the parental genotype Allahabad Safeda and Green Yellow Group (1C) for Arka Kiran. However, traits segregated in hybrid progenies beyond parental ranges, 30 genotypes showed fruit peel colour in the Yellow Group (3–5); while 14 had Green Yellow (1A–1C), four genotypes had Yellow Green (150B), and two hybrids showed peel colour as Yellow Orange (14C) (Fig. 2). The peel colour index values ranged from 69.03 to 85.48, with highest in hybrid GH20_7D and lowest in hybrid GH20_3D. The pulp colour of Allahabad Safeda was white (155D), and Arka Kiran had red pulp colour (39B). Among the hybrid progenies, 45 belonged to the red pulp group (38–49), while 5 hybrids to the white pulp group (NN155A–D).

**Table 3:** Variations in fruit physical parameters among the newly developed guava hybrid progenies and parental genotypes.

S. No	Guava genotype	TCD fruit pulp	TCD fruit peel	Fruit length (cm)	Fruit width (cm)	Fruit length/width	Fruit weight (g)	Fruit thickness of outer pulp (cm)	Fruit diameter of outer calyx (cm)
1	Allahabad Safeda (P_1_)	83.19 ± 1.55^a^	81.71 ± 0.79^a–c^	7.14 ± 0.08^l–q^	5.91 ± 0.1^v–x^	1.21 ± 0.02^b–f^	159.33 ± 8.69^n–r^	1.48 ± 0.09^r–u^	1.08 ± 0.05^g–r^
2	Arka Kiran (P_2_)	64.95 ± 1.28^o–t^	82.36 ± 1.28^ab^	6.94 ± 0.36^n–s^	7.3 ± 0.2^e–m^	0.95 ± 0.07^p^	212.67 ± 3.84^e–k^	2.05 ± 0.08^c–g^	2.47 ± 0.02^a^
3	GH20_1A	72.74 ± 1.44^c–m^	73.9 ± 1.87^h–m^	8.07 ± 0.14^f–k^	7.28 ± 0.39^e–m^	1.12 ± 0.08^e–k^	234 ± 23.52^c–h^	1.87 ± 0.1^e–n^	1.62 ± 0.03^bc^
4	GH20_1B	65.74 ± 1.82^n–t^	73.04 ± 1.07^i–n^	8.67 ± 0.66^b–f^	7.00 ± 0.1^i–p^	1.23 ± 0.08^b–d^	207.33 ± 23.79^f–l^	1.77 ± 0.04^h–p^	0.81 ± 0.03^r–t^
5	GH20_1C	74.36 ± 1.92^b–i^	72.6 ± 1.26^j–n^	7.03 ± 0.02^m–s^	6.86 ± 0.24^j–q^	1.03 ± 0.04^j–p^	170.67 ± 8.09^k–p^	1.48 ± 0.12^q–u^	1.22 ± 0.02^d–m^
6	GH20_1D	68.85 ± 1.17^j–r^	76.65 ± 1.62^d–j^	6.67 ± 0.33^p–t^	6.11 ± 0.10^s–w^	1.09 ± 0.05^h–l^	134.67 ± 6.36^p–r^	1.55 ± 0.02^o–t^	1.02 ± 0.04^j–s^
7	GH20_10D	75.13 ± 2.17^b–f^	82.1 ± 0.92^ab^	8.66 ± 0.06^b–f^	7.43 ± 0.13^d–l^	1.17 ± 0.03^c–h^	240.33 ± 8.69^c–g^	1.88 ± 0.03^e–m^	1.5 ± 0.12^cd^
8	GH20_2B	63.97 ± 1.89^p–t^	74.85 ± 2.36^h–m^	6.78 ± 0.3^p–t^	7.16 ± 0.21^g–n^	0.94 ± 0.01^p^	179 ± 20.52^j–o^	1.92 ± 0.22^d–k^	1.16 ± 0.11^f–p^
9	GH20_2C	66.9 ± 0.58^n–t^	74.92 ± 2.62^h–m^	8.42 ± 0.22^c–g^	6.79 ± 0.09^k–s^	1.24 ± 0.02^bc^	196.67 ± 9.62^h–n^	1.99 ± 0.18^c–h^	1.08 ± 0.04^g–s^
10	GH20_2E	70.79 ± 0.82^e–n^	82.82 ± 1.5^ab^	6.44 ± 0.35^q–t^	6.74 ± 0.36^l–t^	0.96 ± 0.02^op^	167.67 ± 12.33^l–p^	1.82 ± 0.1^f–o^	1.03 ± 0.04^i–s^
11	GH20_3A	63.26 ± 2.05^st^	72.45 ± 1.47^j–n^	9.14 ± 0.26^b^	7.01 ± 0.37^h–p^	1.31 ± 0.04^b^	220 ± 26.1^d–j^	1.87 ± 0.1^e–n^	0.91 ± 0.08^o–t^
12	GH20_3B	69.02 ± 1.35^i–q^	75.09 ± 2.45^h–k^	8.81 ± 0.17^b–e^	7.23 ± 0.16^f–m^	1.22 ± 0.03^b–e^	233.33 ± 3.38^c–h^	1.93 ± 0.12^c–j^	1.07 ± 0.07^h–s^
13	GH20_3C	67.79 ± 2.7^m–t^	82.97 ± 1.13^a^	6.82 ± 0.28^o–s^	6.33 ± 0.03^p–w^	1.08 ± 0.05^h–n^	140 ± 4.73^o–r^	2.13 ± 0.05^b–e^	1.07 ± 0.08^g–s^
14	GH20_3D	66.86 ± 1.97^n–t^	69.03 ± 1.58^n^	6.68 ± 0.16^p–t^	6.16 ± 0.15^q–w^	1.09 ± 0.0^h–m^	134.33 ± 3.18^p–r^	1.74 ± 0.07^h–r^	0.95 ± 0.06^l–s^
15	GH20_4B	66.01 ± 1.3^n–t^	72.83 ± 0.58^i–n^	7.79 ± 0.28^g–l^	7.46 ± 0.53^d–k^	1.05 ± 0.04^i–p^	226 ± 42.44^d–h^	2.2 ± 0.23^a–d^	1.3 ± 0.13^d–i^
16	GH20_5A	64.79 ± 2.29^o–t^	70.38 ± 2.18^mn^	7.17 ± 0.23^l–p^	7.39 ± 0.33^e–m^	0.97 ± 0.05^n–p^	214 ± 23.54^e–j^	1.76 ± 0.06^h–q^	1.31 ± 0.15^d–h^
17	GH20_5C	66.63 ± 2.11^n–t^	76.18 ± 2.25^e–j^	7.17 ± 0.7^l–p^	7.22 ± 0.56^f–m^	1.00 ± 0.1^l–p^	181.67 ± 30.34^i–o^	1.65 ± 0.05^k–t^	1.38 ± 0.22^c–f^
18	GH20_5D	63.59 ± 2.04^r–t^	77.27 ± 1.34^c–i^	8.67 ± 0.07^b–f^	6.05 ± 0.1^t–w^	1.43 ± 0.03^a^	157 ± 1.73^n–r^	1.78 ± 0.11^g–o^	0.91 ± 0.08^n–t^
19	GH20_5E	65.84 ± 1.64^n–t^	72.78 ± 2.07^i–n^	9.02 ± 0.18^bc^	7.00 ± 0.31^i–p^	1.29 ± 0.04^b^	223.67 ± 24.61^d–i^	1.9 ± 0.08^e–l^	1.24 ± 0.06^d–k^
20	GH20_6A	73.36 ± 2.16^c–l^	84.23 ± 1.58^a^	5.59 ± 0.2^u^	5.89 ± 0.22^v–x^	0.95 ± 0.04^p^	135.33 ± 8.57^qr^	1.6 ± 0.22^n–t^	1.3 ± 0.05^d–i^
21	GH20_6B	63.5 ± 2.32^r–t^	83.78 ± 2^a^	7.07 ± 0.07^m–r^	6.98 ± 0.18^i–p^	1.01 ± 0.03^k–p^	196 ± 6.35^h–n^	1.71 ± 0.0^i–s^	1.04 ± 0.12^h–s^
22	GH20_6D	72.93 ± 0.93^c–m^	81.67 ± 1.37^a–c^	8.22 ± 0.13^e–j^	7.55 ± 0.23^d–j^	1.09 ± 0.03^h–l^	249 ± 8.89^b–f^	1.74 ± 0.12^h–r^	1.19 ± 0.16^f–n^
23	GH20_6E	73.6 ± 2.09^c–l^	81.47 ± 0.67^a–c^	7.19 ± 0.29^l–p^	6.7 ± 0.32^m–u^	1.07 ± 0.03^h–n^	167 ± 18.45^l–q^	1.78 ± 0.12^g–o^	0.9 ± 0.05^p–t^
24	GH20_7C	63.73 ± 2.2^q–t^	79.93 ± 1.34^a–g^	8.68 ± 0.15^b–f^	7.89 ± 0.43^c–f^	1.11 ± 0.05^f–k^	268.33 ± 15.86^bc^	2.33 ± 0.03^ab^	1.23 ± 0.13^d–m^
25	GH20_7E	67.56 ± 2.12^m–t^	75.04 ± 1.8^h–l^	7.08 ± 0.12^l–r^	5.74 ± 0.38^wx^	1.25 ± 0.09^bc^	141 ± 17.52^o–r^	1.63 ± 0.14^l–t^	1.48 ± 0.1^c–e^
26	GH20_8A	68.34 ± 1.2^k–s^	75.09 ± 1.96^h–l^	8.22 ± 0.23^e–j^	7.73 ± 0.19^c–h^	1.06 ± 0.01^h–o^	236 ± 11.14^c–h^	1.67 ± 0.14^j–s^	1.07 ± 0.17^h–s^
27	GH20_8B	69.33 ± 1.52^g–p^	80.7 ± 1.11^a–e^	6.42 ± 0.02^r–t^	5.99 ± 0.22^u–w^	1.07 ± 0.04^h–n^	118 ± 9.54^rs^	1.57 ± 0.12^o–t^	0.99 ± 0.14^k–s^
28	GH20_8C	64.03 ± 1.6^p–t^	74.59 ± 1.48^h–m^	7.07 ± 0.07^m–r^	6.4 ± 0.18^o–w^	1.11 ± 0.02^f–k^	161 ± 5.86^m–q^	1.45 ± 0.01^s–u^	0.92 ± 0.02^n–t^
29	GH20_8D	66.5 ± 0.92^n–t^	75.54 ± 0.79^g–k^	8.56 ± 0.06^b–f^	7.65 ± 0.15^c–i^	1.12 ± 0.02^e–k^	245.33 ± 6.69^b–f^	1.97 ± 0.04^c–i^	1.16 ± 0.17^f–p^
30	GH20_8E	68.21 ± 2.01^l–t^	74.14 ± 1.54^h–m^	7.37 ± 0.18^k–p^	6.83 ± 0.16^j–r^	1.08 ± 0.01^h–n^	179.33 ± 7.51^j–o^	1.79 ± 0.13^g–o^	0.87 ± 0.08^q–t^
31	GH20_9A	76.61 ± 2.32^bc^	70.49 ± 1.94^l–n^	6.33 ± 0.2^st^	6.47 ± 0.04^n–v^	0.98 ± 0.05^m–p^	136 ± 2.08^q–s^	1.56 ± 0.11^o–t^	0.88 ± 0.15^p–t^
32	GH20_9B	69.95 ± 3.07^f–o^	76.02 ± 2.93^f–j^	6.91 ± 0.18^n–s^	6.30 ± 0.34^p–w^	1.1 ± 0.04^g–l^	134.33 ± 15.56^p–r^	1.27 ± 0.02^u^	0.65 ± 0.03^t^
33	GH20_10A	69.28 ± 1.53^h–p^	75.98 ± 2.02^f–j^	8.26 ± 0.15^d–i^	6.87 ± 0.23^j–q^	1.2 ± 0.06^b–g^	211.33 ± 8.69^f–k^	2.08 ± 0.14^b–f^	1.79 ± 0.08^b^
34	GH20_11B	71.05 ± 1.77^d–n^	83.71 ± 0.97^a^	7.73 ± 0.3^g–m^	7.19 ± 0.32^f–m^	1.08 ± 0.01^h–n^	202.33 ± 16.9^g–m^	1.87 ± 0.03^e–n^	1.19 ± 0.18^f–o^
35	GH20_11C	76.38 ± 2.07^b–d^	78.37 ± 3.22^b–h^	7.06 ± 0.16^m–r^	6.12 ± 0.15^r–w^	1.15 ± 0.02^c–i^	134.67 ± 3.48^p–r^	1.5 ± 0.08^p–u^	0.97 ± 0.04^k–s^
36	GH20_11D	74.67 ± 2.01^b–h^	84.51 ± 1.66^a^	7.37 ± 0.31^k–p^	7.22 ± 0.18^f–m^	1.02 ± 0.02^j–p^	167 ± 8.08^l–q^	1.6 ± 0.07^n–t^	1 ± 0.11^k–s^
37	GH20_12B	64.87 ± 2.35^o–t^	76.15 ± 2.22^e–j^	9.03 ± 0.27^bc^	7.99 ± 0.14^c–e^	1.13 ± 0.05^d–j^	286.33 ± 20.85^b^	2.08 ± 0.08^b–f^	1.21 ± 0.1^e–m^
38	GH20_12E	75.41 ± 2.7^b–e^	82.11 ± 1.82^ab^	7.34 ± 0.07^l–p^	7.08 ± 0.32^g–o^	1.04 ± 0.04^j–p^	208.67 ± 15.45^f–l^	1.8 ± 0.1^g–o^	1.04 ± 0.05^h–s^
39	GH20_13B	65.81 ± 1.12^n–t^	83.25 ± 1.5^a^	7.52 ± 0.02^j–o^	7.05 ± 0.15^g–o^	1.07 ± 0.02^h–n^	218.33 ± 19.62^d–j^	1.89 ± 0.04^e–m^	1.11 ± 0.1^f–q^
40	GH20_13E	66.51 ± 2.27^n–t^	84.48 ± 2.51^a^	8.33 ± 0.31^c–h^	7.4 ± 0.10^e–m^	1.13 ± 0.04^d–j^	220.33 ± 11.32^d–j^	1.96 ± 0.08^c–i^	1.11 ± 0.01^f–q^
41	GH20_14D	62.83 ± 1.22^t^	73.08 ± 1.99^i–n^	8.76 ± 0.17^b–f^	7.18 ± 0.09^f–n^	1.22 ± 0.01^b–e^	223.67 ± 5.36^d–i^	1.85 ± 0.06^e–n^	0.9 ± 0.06^p–t^
42	GH20_15B	65.25 ± 1.21^o–t^	76.75 ± 1.87^d–j^	8.61 ± 0.09^b–f^	7.63 ± 0.21^c–i^	1.13 ± 0.02^d–j^	230.33 ± 8.84^c–h^	1.78 ± 0.06^g–o^	1.14 ± 0.06^f–q^
43	GH20_15E	73.61 ± 2.04^c–k^	80.31 ± 1.26^a–f^	8.63 ± 0.34^b–f^	8.33 ± 0.44^bc^	1.04 ± 0.02^j–p^	227.67 ± 16.15^c–h^	1.87 ± 0.06^e–n^	1.31 ± 0.17^d–h^
44	GH20_18B	74.24 ± 1.38^b–j^	84.37 ± 1.75^a^	7.61 ± 0.2^i–n^	7.35 ± 0.19^e–m^	1.04 ± 0.00^j–p^	203 ± 3.61^g–m^	1.56 ± 0.09^o–t^	1.22 ± 0.16^d–m^
45	GH20_16D	75.29 ± 2.19^b–f^	83.86 ± 1.98^ba^	7.69 ± 0.39^h–l^	7.45 ± 0.43^d–l^	1.03 ± 0.01^j–p^	178.33 ± 8.41^j–o^	1.73 ± 0.04^h–s^	1.31 ± 0.05^d–h^
46	GH20_17B	73.43 ± 2.14^c–l^	71.04 ± 1.17^k–n^	8.67 ± 0.17^b–f^	8.14 ± 0.31^b–d^	1.07 ± 0.02^h–n^	260.33 ± 11.26^b–d^	1.9 ± 0.08^e–l^	1.29 ± 0.08^d–j^
47	GH20_17E	74.69 ± 2.71^b–g^	74.68 ± 1.98^h–m^	6.1 ± 0.39^tu^	5.21 ± 0.01^x^	1.17 ± 0.07^c–h^	79.33 ± 4.7^s^	1.38 ± 0.03^tu^	0.95 ± 0.04^m–s^
48	GH20_5B	82.90 ± 1.74^a^	83.47 ± 1.25^a^	8.59 ± 0.36^b–f^	7.77 ± 0.21^c–g^	1.11 ± 0.02^f–k^	254.67 ± 14.24^b–e^	1.9 ± 0.04^e–l^	0.8 ± 0.02^st^
49	GH20_11A	75.46 ± 2.37^b–e^	81.22 ± 2.69^a–d^	8.95 ± 0.2^b–d^	9.44 ± 0.28^a^	0.95 ± 0.01^p^	406.33 ± 16.9^a^	2.46 ± 0.09^a^	1.48 ± 0.15^c–e^
50	GH20_12C	79.55 ± 1.82^ab^	83.93 ± 1.82^ba^	10.13 ± 0.28^a^	8.79 ± 0.15^ab^	1.16 ± 0.05^c–i^	381.33 ± 15.41^a^	2.21 ± 0.06^a–c^	1.35 ± 0.13^c–g^
51	GH20_18A	74.39 ± 2.22^b–i^	80.07 ± 1.87^a–g^	7.04 ± 0.04^m–s^	7.22 ± 0.04^f–m^	0.97 ± 0.0^n–p^	142.67 ± 1.76^o–r^	1.61 ± 0.03^m–t^	1.13 ± 0.02^f–q^
52	GH20_20B	69.57 ± 2.60^g–o^	80.37 ± 0.99^a–f^	8.28 ± 0.21^d–i^	8.34 ± 0.24^bc^	1 ± 0.01^l–p^	218.67 ± 6.89^d–j^	1.79 ± 0.07^g–o^	1.23 ± 0.05^d–l^
	Range	62.83–83.19	69.03–84.51	5.59–10.13	5.21–9.44	0.95–1.43	79.33–406.33	1.27–2.46	0.65–2.47
	Mean	70.06	78.04	7.74	7.08	1.09	201.04	1.80	1.16
	LSD (*p* ≤ 0.05)	5.40	4.60	0.71	0.71	0.11	42.20	0.27	0.28
	CV (%)	7.29	5.85	12.34	11.26	9.39	29.47	13.19	24.64

Values are mean ± SE. Means followed by different superscript letters within a column represent sampling significant difference.

The fruit traits among the evaluated guava hybrid progenies varied considerably, with length ranging from 5.59 to 10.13 cm, width from 5.21 to 9.44 cm, and weight from 79.33 to 406.33 g. In contrast, the parental genotype Allahabad Safeda recorded values of 7.14 cm, 5.91 cm, and 159.33 g for fruit length, width, and weight, respectively, while Arka Kiran had 6.94 cm, 7.30 cm, and 212.67 g, respectively. Among the hybrid progenies, GH20_12C had the longest fruit (10.13 cm), while it was the shortest (5.59 cm) in GH20_6A. The highest fruit width was observed for the hybrid GH20_11A (9.44 cm), followed by GH20_12C (8.79 cm), whereas the minimum fruit width (5.21 cm) was noted for the hybrid, GH20_17E. The highest fruit length/width ratio was recorded in hybrid GH20_5D (1.43), and the lowest was in GH20_11A (0.95) (Table 3). The heaviest fruit was registered in GH20_11A (406.33 g), while the lightest was in GH20_17E (79.33 g), showing a five-fold variation.

The pulp thickness of mature fruits was measured as 1.48 cm in Allahabad Safeda and 2.05 cm in Arka Kiran, while in the hybrids it varied from 1.27 to 2.46 cm. It was observed to be the highest in hybrid GH20_11A (2.46 cm), followed by GH20_7C (2.33 cm), while the lowest (1.27 cm) was in GH20_9B. The calyx end cavity diameter of fruit was recorded as highest in Arka Kiran (2.47 cm), followed by GH20_10A (1.79 cm), both classified in the large group (> 1 cm), while the smallest (0.65 cm) cavity at the calyx end was observed in hybrid GH20_9B.

### Fruit biochemical parameters

**Fig. 3:**
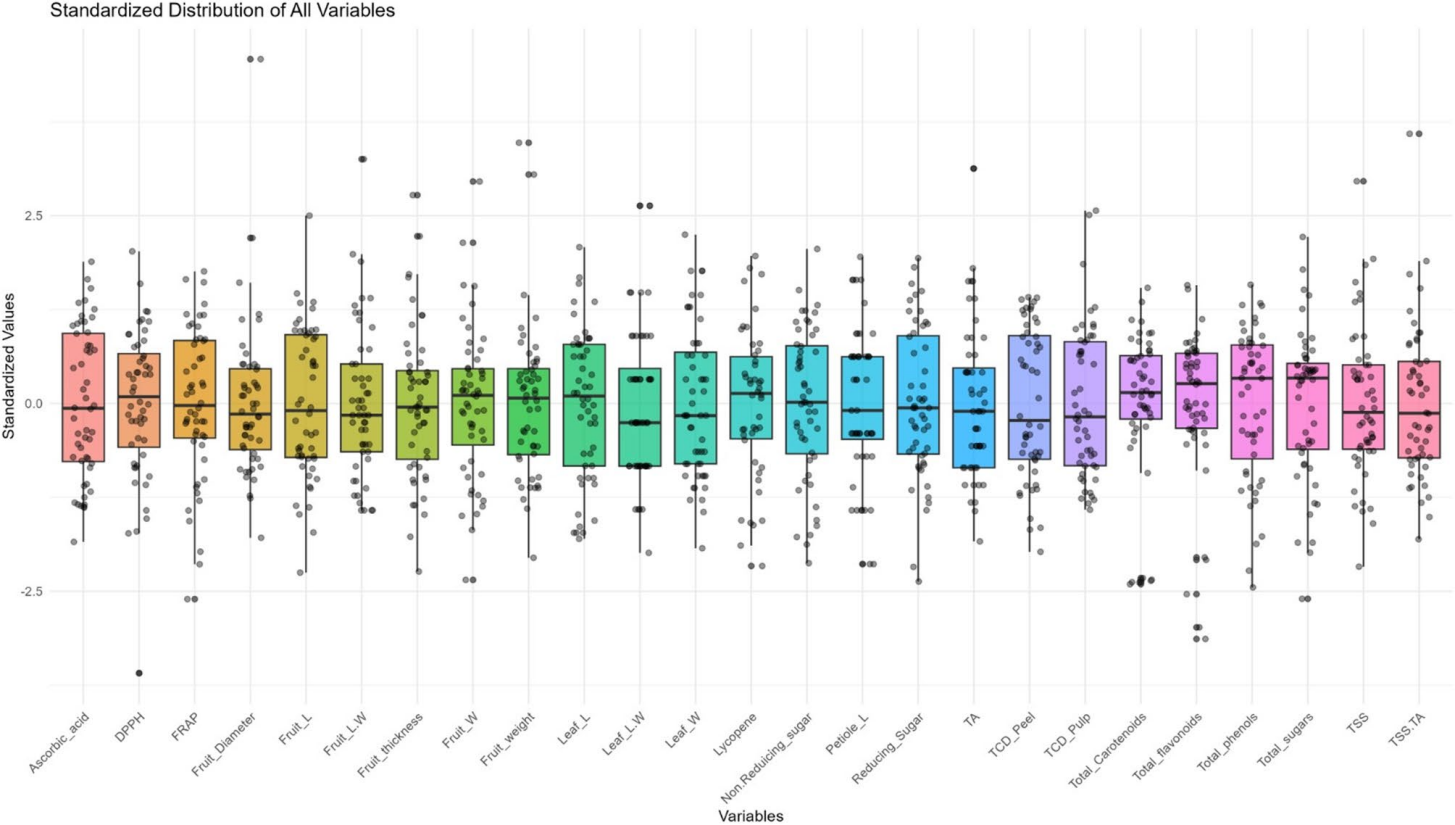
Boxplot showing the standardized distribution of evaluated parameters among the newly developed guava hybrid progenies and their parents. [The boxplot displays standardized values (z-scores) of morpho-biochemical parameters in studied guava genotypes. Each box represents the interquartile range, with the horizontal line indicating the median, whiskers extending to the most typical minimum and maximum values, and excluding points representing outliers. Most variables are standardized around zero, with some parameters like ascorbic acid, fruit length, leaf length, leaf width, reducing sugar, TCD peel, TCD pulp and total phenol exhibiting wider variation, while others display relatively compact distributions. Moreover, parameters like fruit diameter, fruit length width ratio, pulp thickness, fruit width, fruit weight, leaf length width ratio, TA, TSS and TSS/TA ratio had several higher outliers beyond whiskers, representing exceptional genotypes with higher value. In contrast, other traits like DPPH, FRAP, fruit width, total carotenoids, total flavonoids and total sugars had several lower outliers beyond whiskers indicating presence of exceptional genotypes with lower value].

The guava hybrid progenies and their parental genotypes exhibited substantial variations in the assayed fruit biochemical parameters encompassing both quality traits and antioxidants (Tables 4 and 5), with their distribution patterns depicted in Fig. 3. The pulp total soluble solids (TSS) content in hybrid progenies varied from 10.33 to 14.80°Brix compared to 11.60°Brix in Allahabad Safeda and 12.00°Brix in Arka Kiran. The hybrid, GH20_3A, had the highest TSS value, while lowest was recorded in hybrid GH20_18A. The highest titratable acidity was present in GH20_6A (1.11%), followed by GH20_18A (0.85%), whereas the lowest was in GH20_5C (0.25%). The parental genotype, Allahabad Safeda, recorded titratable acidity of 0.57%, while Arka Kiran showed a lower value (0.32%). The results showed that the studied guava hybrids and parental genotypes had higher reducing sugar content than non-reducing sugar content. The total, reducing, and non-reducing sugars contents in hybrid progenies varied from 6.39 to 9.61%, 3.79 to 5.56%, and 2.51 to 4.19%, respectively. However, the total, reducing, and non-reducing sugars contents of the parental genotype, Allahabad Safeda were 8.08, 4.31, and 3.76%, respectively, while Arka Kiran had 8.41, 4.74, and 3.67%, respectively. The highest total sugars content was recorded in hybrid GH20_3A (9.61%), which was comparable to that in GH20_8D (9.32%), while the lowest in GH20_18A (6.39%). The reducing sugars content was estimated to be highest in hybrid GH20_8D (5.56%), followed by GH20_12B (5.51%), while lowest in GH20_18A (3.79%). The highest non-reducing sugars content was recorded in GH20_3A (4.19%), and the minimum was in GH20_4B (2.51%). The ascorbic acid content in the fruits of the Allahabad Safeda was recorded as 212.27 mg/100 g, while Arka Kiran had 279.67 mg/100 g; however, it varied from 65.13 to 302.20 mg/100 g among hybrid progenies. The white pulp guava hybrid GH20_11A exhibited the highest ascorbic acid content (302.20 mg/100 g), whereas the pink pulp hybrid GH20_17E had the lowest ascorbic acid content (65.13 mg/100 g).

**Table 4:** Variations in fruit biochemical parameters among the newly developed guava hybrid progenies and parental genotypes.

S. No	Guava genotype	TSS (°B)	Titratable acidity (%)	TSS/ Acid ratio	Ascorbic acid (mg/100g)	Lycopene (mg/100 g)	Total carotenoids (mg/100 g)
1	Allahabad Safeda (P_1_)	11.6 ± 0.35^o–v^	0.57 ± 0.04^d–h^	20.56 ± 1.73^h–q^	212.27 ± 3.69^ij^	0.00 ± 0.00^u^	0.78 ± 0.01^w^
2	Arka Kiran (P_2_)	12.00 ± 0.23^i–q^	0.32 ± 0.02^ij^	38.03 ± 1.60^a–c^	279.67 ± 1.45^a–c^	8.7 ± 0.06^ab^	9.56 ± 0.15^a^
3	GH20_1A	11.50 ± 0.29^p–w^	0.47 ± 0.04^f–i^	25.01 ± 1.99^d–p^	131.53 ± 3.91^s–v^	5.13 ± 0.34^kl^	6.49 ± 0.33^i–q^
4	GH20_1B	11.40 ± 0.31^q–w^	0.47 ± 0.12^f–i^	29.02 ± 9.56^b–l^	249.8 ± 17.66^d–g^	6.63 ± 0.21^d–f^	8.2 ± 0.35^b–e^
5	GH20_1C	11.73 ± 0.27^m–s^	0.55 ± 0.04^d–h^	21.52 ± 2.01f.^–q^	102.83 ± 5.19^x^	4.33 ± 0.27^mn^	8.04 ± 0.02^c–f^
6	GH20_1D	11.80 ± 0.31^m–r^	0.60 ± 0.04^c–g^	20.06 ± 1.97^h–q^	107.67 ± 9.47^wx^	4.28 ± 0.21^mn^	6.86 ± 0.11^h–n^
7	GH20_10D	12.17 ± 0.34^g–p^	0.68 ± 0.08^b–f^	18.48 ± 2.04^i–q^	136.67 ± 6.24^p–u^	4.12 ± 0.28^mn^	6.38 ± 0.29^j–r^
8	GH20_2B	12.17 ± 0.60^g–p^	0.64 ± 0.07^b–g^	19.83 ± 3.19^h–q^	231 ± 10.97^f–i^	5.6 ± 0.16^h–k^	7.53 ± 0.22^d–i^
9	GH20_2C	12.33 ± 0.33^f–n^	0.76 ± 0.15^b–d^	17.47 ± 3.40^l–q^	144.8 ± 6.72^p–t^	4.62 ± 0.15^lm^	6.51 ± 0.16^i–q^
10	GH20_2E	12.03 ± 0.32^i–q^	0.38 ± 0.08^h–j^	34.57 ± 7.38^b–e^	156.67 ± 6.49^n–r^	6.04 ± 0.39^f–i^	7.27 ± 0.39^e–k^
11	GH20_3A	14.80 ± 0.20^a^	0.55 ± 0.04^d–h^	27.03 ± 1.83^b–o^	269.47 ± 9.79^b–d^	5.40 ± 0.04^jk^	6.77 ± 0.23^h–o^
12	GH20_3B	12.53 ± 0.29^f–k^	0.55 ± 0.04^d–h^	22.98 ± 2.13^e–q^	178.6 ± 5.95^k–n^	5.80 ± 0.30^g–j^	6.92 ± 0.92^g–m^
13	GH20_3C	11.70 ± 0.15^m–t^	0.47 ± 0.04^f–i^	25.63 ± 2.98^c–o^	153.47 ± 3.01^o–s^	4.28 ± 0.29^mn^	6.32 ± 0.32^k–r^
14	GH20_3D	12.50 ± 0.29^f–l^	0.55 ± 0.04^d–h^	22.78 ± 1.27^e–q^	94.87 ± 3.62^x^	1.78 ± 0.09^r^	8.22 ± 0.13^b–e^
15	GH20_4B	11.00 ± 0.58^u–x^	0.81 ± 0.11^bc^	14.34 ± 2.58^o–q^	267.43 ± 7.48^b–d^	5.71 ± 0.05^g–k^	7.41 ± 0.08^e–j^
16	GH20_5A	12.00 ± 0.29^i–q^	0.85 ± 0.04^b^	14.22 ± 0.48^o–q^	287.33 ± 3.10^ab^	9.03 ± 0.04^a^	9.13 ± 0.04^ab^
17	GH20_5C	12.67 ± 0.44^f–i^	0.25 ± 0.00^j^	50.67 ± 1.76^a^	169.63 ± 4.80^l–n^	0.53 ± 0.03^tu^	4.07 ± 0.06^v^
18	GH20_5D	10.97 ± 0.15^u–x^	0.64 ± 0.07^b–g^	17.64 ± 1.82^k–q^	96.87 ± 2.86^x^	4.27 ± 0.35^mn^	5.71 ± 0.28^p–u^
19	GH20_5E	11.20 ± 0.12^r–w^	0.38 ± 0.08^h–j^	32.03 ± 6.66^b–h^	148.03 ± 4.56^o–t^	6.43 ± 0.15^ef^	5.44 ± 0.49^r–u^
20	GH20_6A	11.03 ± 0.12^t–w^	1.11 ± 0.09^a^	10.10 ± 0.83^q^	225.67 ± 7.62^hi^	5.21 ± 0.14^j–l^	5.26 ± 0.03^s–u^
21	GH20_6B	11.67 ± 0.33^n–u^	0.38 ± 0.08^h–j^	33.72 ± 7.7^b–g^	194.13 ± 7.08^jk^	6.31 ± 0.19^fg^	7.57 ± 0.14^c–h^
22	GH20_6D	12.37 ± 0.3^f–m^	0.68 ± 0.04^b–f^	18.3 ± 1.03^i–q^	96.67 ± 3.53^x^	1.10 ± 0.25^st^	5.02 ± 0.25^t–v^
23	GH20_6E	12.73 ± 0.37^e–h^	0.42 ± 0.04^g–j^	30.56 ± 2.46^b–k^	186.8 ± 17.2^kl^	5.53 ± 0.18^i–k^	6.06 ± 0.77^l–s^
24	GH20_7C	11.77 ± 0.29^m–r^	0.64 ± 0.07^b–g^	18.89 ± 1.78^i–q^	255.17 ± 4.94^de^	7.10 ± 0.14^cd^	7.38 ± 0.24^e–j^
25	GH20_7E	12.27 ± 0.37^g–o^	0.81 ± 0.11^bc^	15.87 ± 2.47^m–q^	177.67 ± 11.47^k–n^	1.70 ± 0.44^rs^	6.00 ± 0.43^l–t^
26	GH20_8A	12.50 ± 0.29^f–l^	0.60 ± 0.04^c–g^	21.19 ± 1.72^g–q^	98.40 ± 3.360^x^	7.58 ± 0.31^c^	5.84 ± 0.47^n–u^
27	GH20_8B	11.77 ± 0.15^m–r^	0.42 ± 0.09^g–j^	30.89 ± 7.56^b–j^	114.07 ± 3.52^u–x^	2.88 ± 0.52^pq^	5.99 ± 0.97^l–t^
28	GH20_8C	13.63 ± 0.45^bc^	0.64 ± 0.07^b–g^	21.84 ± 1.9^e–q^	241.33 ± 9.39^e–h^	1.77 ± 0.10^r^	7.90 ± 0.49^c–g^
29	GH20_8D	13.83 ± 0.44^b^	0.47 ± 0.09^f–i^	31.24 ± 4.36^b–i^	248.07 ± 7.98^d–h^	5.64 ± 0.47^h–k^	6.41 ± 0.15^j–r^
30	GH20_8E	11.67 ± 0.17^n–u^	0.42 ± 0.09^g–j^	30.69 ± 7.66^b–j^	95.67 ± 1.12^x^	3.98 ± 0.41^no^	5.81 ± 0.71^o–u^
31	GH20_9A	11.57 ± 0.26^p–v^	0.64 ± 0.07^b–g^	18.70 ± 2.35^i–q^	127.93 ± 4.99^t–w^	4.17 ± 0.27^mn^	7.71 ± 0.59^c–h^
32	GH20_9B	12.50 ± 0.17^f–l^	0.47 ± 0.09^f–i^	28.44 ± 4.48^b–m^	227.50 ± 6.29^g–i^	7.02 ± 0.44^c–e^	8.55 ± 0.13^a–d^
33	GH20_10A	11.87 ± 0.13^k–r^	0.72 ± 0.04^b–e^	16.60 ± 1.09^l–q^	133.67 ± 5.78^r–v^	2.55 ± 0.34^q^	5.39 ± 0.30^r–u^
34	GH20_11B	13.40 ± 0.15^b–e^	0.51 ± 0.08^e–i^	27.56 ± 4.37^b–n^	262.00 ± 5.29^c–e^	6.60 ± 0.23^d–f^	7.96 ± 0.02^c–f^
35	GH20_11C	11.83 ± 0.17^l–r^	0.34 ± 0.04^ij^	36.61 ± 5.71^b–d^	231.40 ± 18.82^f–i^	3.37 ± 0.26^op^	5.96 ± 0.29^m–t^
36	GH20_11D	12.60 ± 0.12^f–j^	0.51 ± 0.15^e–i^	30.52 ± 10.17^b–k^	251.70 ± 5.86^d–f^	3.08 ± 0.21^pq^	5.54 ± 0.22^q–u^
37	GH20_12B	13.90 ± 0.06^b^	0.38 ± 0.08^h–j^	39.74 ± 8.17^ab^	227.20 ± 6.25^g–i^	6.14 ± 0.38^f–h^	7.48 ± 0.56^e–i^
38	GH20_12E	12.97 ± 0.09^c–f^	0.72 ± 0.04^b–e^	18.12 ± 0.94^j–q^	112.47 ± 3.43^v–x^	5.55 ± 0.35^h–k^	4.81 ± 0.25^uv^
39	GH20_13B	13.00 ± 0.17^c–f^	0.42 ± 0.04^g–j^	31.24 ± 2.59^b–i^	94.23 ± 17.72^x^	5.30 ± 0.10^jk^	6.68 ± 0.20^h–p^
40	GH20_13E	12.57 ± 0.35^f–j^	0.47 ± 0.12^f–i^	32.02 ± 10.52^b–h^	135.67 ± 6.41^q–v^	1.65 ± 0.30^rs^	5.80 ± 0.39^o–u^
41	GH20_14D	13.43 ± 0.12^b–d^	0.51 ± 0.08^e–i^	27.61 ± 4.33^b–n^	180.60 ± 10.88^k–m^	3.22 ± 0.13^p^	8.22 ± 0.06^b–e^
42	GH20_15B	10.83 ± 0.09^wx^	0.42 ± 0.04^g–j^	26.12 ± 2.57^c–o^	136.80 ± 14.46^p–u^	5.62 ± 0.30^h–k^	6.37 ± 0.78^j–r^
43	GH20_15E	11.97 ± 0.15^j–q^	0.64 ± 0.07^b–g^	19.26 ± 2.07^h–q^	158.00 ± 3.06^m–q^	4.32 ± 0.12^mn^	7.02 ± 0.50^f–l^
44	GH20_18B	12.07 ± 0.18^h–q^	0.38 ± 0.08^h–j^	34.36 ± 6.75^b–f^	200.00 ± 5.68^jk^	8.53 ± 0.04^ab^	7.69 ± 0.12^c–h^
45	GH20_16D	11.83 ± 0.44^l–r^	0.59 ± 0.11^c–h^	21.74 ± 4.93^e–q^	152.00 ± 5.09^o–s^	7.08 ± 0.32^cd^	7.40 ± 0.47^e–j^
46	GH20_17B	13.00 ± 0.12^c–f^	0.47 ± 0.04^f–i^	28.44 ± 3.15^b–m^	215.53 ± 9.92^ij^	8.34 ± 0.17^b^	8.60 ± 0.10^a–c^
47	GH20_17E	12.53 ± 0.20^f–k^	0.72 ± 0.04^b–e^	17.55 ± 1.31^k–q^	65.13 ± 4.16^y^	5.22 ± 0.39^j–l^	6.08 ± 0.42^l–s^
48	GH20_5B	12.77 ± 0.12^d–g^	0.85 ± 0.09^b^	15.38 ± 1.47^n–q^	251.33 ± 22.64^d–f^	0.00 ± 0.00^u^	0.84 ± 0.04^w^
49	GH20_11A	13.50 ± 0.15^bc^	0.59 ± 0.08^c–h^	23.5 ± 2.67^e–p^	302.20 ± 6.32^a^	0.00 ± 0.00^u^	0.91 ± 0.03^w^
50	GH20_12C	12.67 ± 0.12^f–i^	0.42 ± 0.12^g–j^	34.44 ± 8.64^b–f^	242.33 ± 3.28^e–h^	0.00 ± 0.00^u^	0.96 ± 0.02^w^
51	GH20_18A	10.33 ± 0.18^x^	0.85 ± 0.09^b^	12.48 ± 1.34^pq^	256.47 ± 6.48^c–e^	0.00 ± 0.00^u^	0.83 ± 0.02^w^
52	GH20_20B	11.07 ± 0.07^s–w^	0.34 ± 0.09ij	36.65 ± 7.35^b–d^	159.60 ± 4.66^m–p^	0.00 ± 0.00^u^	0.88 ± 0.02^w^
	Range	10.33–14.80	0.25–1.11	10.1–50.67	65.13–302.20	0.00–9.03	0.78–9.56
	Mean	12.22	0.55	23.23	182.23	4.40	6.13
	LSD (*p* ≤ 0.05)	0.68	0.21	13.02	23.29	0.61	1.03
	CV (%)	7.13	30.41	31.96	34.86	41.33	36.28

Values are mean ± SE. Means followed by different superscript letters within a column represent sampling significant differences.

**Table 5:** Variations in antioxidant and sugar profiles among the newly developed guava hybrid progenies and parental genotypes.

S. No	Guava genotype	Total Phenols (mg GAE/100g)	Total Flavonoids (mg QE/100g)	DPPH-radical scavenging activity (%)	FRAP (µmol Trolox/g)	Total sugars (%)	Reducing sugars (%)	Non-reducing sugars (%)
1	Allahabad Safeda(P_1_)	340.77 ± 1.87^ab^	154.53 ± 1.22^a^	93.73 ± 0.81^a–c^	15.62 ± 0.3^k–p^	8.08 ± 0.08^i–l^	4.31 ± 0.07^r–v^	3.76 ± 0.02^b–f^
2	Arka Kiran (P_2_)	348.71 ± 0.99^a^	145.93 ± 2.8^a–c^	94.96 ± 0.07^a^	20.69 ± 0.53^a–d^	8.41 ± 0.24^e–i^	4.74 ± 0.08^i–m^	3.67 ± 0.16^b–i^
3	GH20_1A	286.4 ± 2.89^o–q^	130.97 ± 2.88^c–o^	89.51 ± 0.47^t–v^	10.36 ± 0.53^uv^	8.53 ± 0.2^e–h^	5.07 ± 0.15^e–g^	3.47 ± 0.07^f–n^
4	GH20_1B	284.17 ± 11.29^o–q^	138.73 ± 1.27^a–f^	91.15 ± 0.3^j–s^	16.65 ± 0.1^h–n^	7.23 ± 0.09^p–r^	3.87 ± 0.06^w^	3.37 ± 0.04^i–p^
5	GH20_1C	308.57 ± 3.86^j–l^	123.94 ± 4.64^e–q^	90.81 ± 1.01^m–u^	12.58 ± 0.53^r–u^	8.33 ± 0.15f.^–j^	4.52 ± 0.19^l–s^	3.81 ± 0.07^b–e^
6	GH20_1D	240.37 ± 2.53^v^	130.22 ± 3.79^c–p^	92.45 ± 0.62^c–l^	13.86 ± 0.1^o–s^	7.8 ± 0.06^k–n^	4.25 ± 0.08^t–v^	3.55 ± 0.02^e–m^
7	GH20_10D	323.57 ± 1.54^c–h^	85.27 ± 5.8^r^	92.7 ± 0.42^c–j^	18.85 ± 1.41^c–h^	8.43 ± 0.07^e–i^	4.75 ± 0.08^i–l^	3.68 ± 0.03^b–h^
8	GH20_2B	334.47 ± 3.09^bc^	122.2 ± 2.05^g–q^	89.25 ± 0.39^uv^	15.86 ± 2.97^k–o^	8.74 ± 0.27^c–e^	4.94 ± 0.14^f–i^	3.8 ± 0.15^b–e^
9	GH20_2C	314.77 ± 2.17^f–k^	137.61 ± 1.84^b–h^	92.19 ± 0.63^c–n^	13.55 ± 0.27^p–s^	7.75 ± 0.27^l–o^	4.44 ± 0.12^o–u^	3.32 ± 0.15^k–q^
10	GH20_2E	324.37 ± 4.36^c–g^	118.21 ± 3.46^l–q^	93 ± 0.76^b–i^	19.7 ± 1.21^c–f^	8.47 ± 0.2^e–i^	4.74 ± 0.25^i–m^	3.73 ± 0.08^b–g^
11	GH20_3A	321.93 ± 2.49^d–i^	133.67 ± 5.86^c–n^	91.71 ± 1.76^f–r^	22.01 ± 0.26^ab^	9.61 ± 0.02^a^	5.42 ± 0.08^a–c^	4.19 ± 0.07^a^
12	GH20_3B	323.77 ± 2.64^c–g^	138.43 ± 1.19^a–g^	93.74 ± 0.38^a–c^	20.64 ± 0.17^a–d^	8.39 ± 0.16^e–i^	4.52 ± 0.17^l–s^	3.86 ± 0.05^b–d^
13	GH20_3C	281.1 ± 2.34^pq^	117.27 ± 2.26^n–q^	92.36 ± 0.66^c–m^	19.44 ± 0.45^c–g^	7.95 ± 0.05^j–m^	4.55 ± 0.18^k–r^	3.4 ± 0.14^h–o^
14	GH20_3D	284.33 ± 3.93^o–q^	136.52 ± 2.13^c–j^	92.62 ± 0.91^c–k^	22.13 ± 0.34^ab^	8.34 ± 0.11^f–j^	4.78 ± 0.06^i–k^	3.56 ± 0.11^d–m^
15	GH20_4B	225.67 ± 4.91^wx^	68.27 ± 1.35^s^	91.8 ± 0.57^e–q^	16.26 ± 0.72^j–n^	6.80 ± 0.07^s^	4.29 ± 0.02^s–v^	2.51 ± 0.06^y^
16	GH20_5A	322.5 ± 2.78^c–i^	139.95 ± 2.48^a–e^	92.83 ± 0.78^b–i^	16.26 ± 0.11^i–n^	7.48 ± 0.09^n–q^	4.67 ± 0.1^j–p^	2.81 ± 0.09^u–y^
17	GH20_5C	325.5 ± 3.4^c–f^	129.5 ± 3.87^c–p^	91.5 ± 0.94^h–r^	17.48 ± 0.17^f–m^	8.42 ± 0.24^e–i^	5.15 ± 0.09^d–f^	3.27 ± 0.16^l–r^
18	GH20_5D	268.33 ± 3.28^rs^	118.03 ± 1.16^m–q^	92.1 ± 0.52^d–o^	18.53 ± 0.73^d–i^	6.89 ± 0.05^rs^	4.22 ± 0.02^uv^	2.66 ± 0.07^w–y^
19	GH20_5E	339.67 ± 3.94^ab^	123.67 ± 4.64^e–q^	90.59 ± 0.75^o–v^	16.18 ± 0.82^j–n^	7.24 ± 0.09^p–r^	4.5 ± 0.14^l–t^	2.74 ± 0.05^v–y^
20	GH20_6A	301.53 ± 2.94^l–n^	137.4 ± 1.32^b–i^	91.11 ± 1.23^k–s^	19.64 ± 1.43^c–g^	7.14 ± 0.09^q–s^	4.49 ± 0.11^m–t^	2.65 ± 0.04^w–y^
21	GH20_6B	318.5 ± 4.16^e–j^	123.22 ± 4.77^f–q^	93.57 ± 0.48^a–d^	21.12 ± 0.78^a–c^	8.18 ± 0.06^h–k^	5.13 ± 0.02^d–f^	3.06 ± 0.05^q–u^
22	GH20_6D	311.23 ± 3.57^i–l^	85.9 ± 1.22^r^	93.53 ± 0.56^a–d^	20.3 ± 0.5^a–e^	8.43 ± 0.12^e–i^	5.22 ± 0.06^c–e^	3.21 ± 0.06^n–s^
23	GH20_6E	255.67 ± 7.37^tu^	116.07 ± 2.31^o–q^	91.5 ± 0.75^h–r^	15.7 ± 1.47^k–p^	8.4 ± 0.18^e–i^	4.79 ± 0.06^h–k^	3.61 ± 0.12^c–k^
24	GH20_7C	312.73 ± 2.4^g–l^	130.33 ± 1.83^c–p^	91.62 ± 0.9^g–r^	15.72 ± 0.82^k–p^	7.69 ± 0.2^l–o^	4.79 ± 0.02^h–k^	2.9 ± 0.19^t–x^
25	GH20_7E	322 ± 2.75^d–i^	134.67 ± 2.35^c–l^	91.02 ± 0.68^l–t^	17.85 ± 0.52^f–k^	8.37 ± 0.16^e–i^	5.2 ± 0.05^c–e^	3.17 ± 0.12^n–t^
26	GH20_8A	313.1 ± 2.31^g–l^	124.5 ± 2.57^d–p^	93.14 ± 0.54^b–g^	20.18 ± 1.07^b–e^	8.47 ± 0.13^e–i^	5.21 ± 0.07^c–e^	3.26 ± 0.07^m–r^
27	GH20_8B	315.33 ± 3.47^f–k^	117.59 ± 2.92^m–q^	92.78 ± 0.64^b–i^	15.28 ± 0.25^m–q^	7.77 ± 0.16^l–o^	4.7 ± 0.06^i–n^	3.08 ± 0.11^p–u^
28	GH20_8C	292.07 ± 2.6^n–p^	142.33 ± 1.86^a–c^	93.27 ± 0.46^b–f^	20.65 ± 0.24^a–d^	8.97 ± 0.07^b–d^	5.38 ± 0.09^a–d^	3.59 ± 0.04^c–k^
29	GH20_8D	328.87 ± 3.89^b–e^	153.5 ± 37.02^ab^	91.88 ± 0.72^e–p^	16.81 ± 0.7^h–n^	9.32 ± 0.21^b^	5.56 ± 0.05^a^	3.76 ± 0.16^b–f^
30	GH20_8E	275.6 ± 2.67^qr^	133.91 ± 2.63^c–m^	89.21 ± 0.39^v^	10.88 ± 0.25^t–v^	7.79 ± 0.2^k–o^	4.43 ± 0.09^o–v^	3.36 ± 0.2^j–p^
31	GH20_9A	311.6 ± 3.16^h–l^	65.34 ± 3.1^s^	93.26 ± 0.37^b–f^	17.76 ± 1.25^f–k^	7.58 ± 0.03^m–p^	4.4 ± 0.02^q–v^	3.18 ± 0.02^n–t^
32	GH20_9B	332.3 ± 2.34^b–d^	121.6 ± 4.97^h–q^	92.58 ± 0.69^c–l^	18.89 ± 0.77^c–h^	8.33 ± 0.14^f–j^	4.86 ± 0.03^g–j^	3.46 ± 0.16^f–n^
33	GH20_10A	253.5 ± 13.65^u^	134.83 ± 3.67^c–k^	92.45 ± 0.44^c–l^	17.03 ± 0.5^h–m^	8.29 ± 0.12^g–j^	4.86 ± 0.08^g–j^	3.43 ± 0.2^g–n^
34	GH20_11B	267.1 ± 2.46^r–t^	132.3 ± 2.82^c–o^	93.05 ± 0.84^b–h^	18.32 ± 0.96^e–j^	8.63 ± 0.09^d–g^	5.27 ± 0.04^b–e^	3.36 ± 0.05^j–p^
35	GH20_11C	308.4 ± 3.7^j–l^	114.41 ± 1.31^pq^	92.49 ± 0.99^c–l^	17.38 ± 0.99^g–m^	7.87 ± 0.33^k–n^	4.63 ± 0.12^j–q^	3.23 ± 0.23^n–s^
36	GH20_11D	294.02 ± 3.34^m–o^	120.15 ± 3.3^j–q^	93.53 ± 0.54^a–d^	17.75 ± 0.21^f–k^	8.38 ± 0.14^e–i^	4.48 ± 0.14^n–t^	3.89 ± 0.01^bc^
37	GH20_12B	285.9 ± 2.48^o–q^	137.63 ± 2.07^b–h^	90.2 ± 1.01^r–v^	13.65 ± 0.4^o–s^	9.09 ± 0.02^bc^	5.51 ± 0.04^b^	3.58 ± 0.02^d–l^
38	GH20_12E	218.5 ± 3.06^x^	107.8 ± 1.9^q^	86.36 ± 1.02^w^	8.92 ± 0.07^v^	8.68 ± 0.14^d–g^	5.04 ± 0.05^e–h^	3.64 ± 0.1^c–j^
39	GH20_13B	264.47 ± 2.79^r–u^	122.03 ± 3.43^g–q^	91.58 ± 1.05^g–r^	15.44 ± 1.3^l–q^	8.53 ± 0.11^e–h^	4.68 ± 0.06^j–n^	3.85 ± 0.05^b–e^
40	GH20_13E	284.2 ± 3.68^o–q^	76.64 ± 0.^97rs^	89.68 ± 0.82^s–v^	19.64 ± 0.49^c–g^	8.44 ± 0.03^e–i^	4.64 ± 0.04^j–q^	3.8 ± 0.02^b–e^
41	GH20_14D	315.03 ± 4.56^f–k^	114.2 ± 3.03^pq^	92.45 ± 0.78^c–l^	19.6 ± 0.3^c–g^	8.56 ± 0.09^e–h^	5.19 ± 0.04^c–f^	3.37 ± 0.05^i–p^
42	GH20_15B	262.03 ± 2.67^s–u^	140.09 ± 2.13^a–e^	92.49 ± 0.71^c–l^	17.67 ± 1.08^f–l^	6.89 ± 0.15^rs^	4.18 ± 0.04^v^	2.71 ± 0.11^v–y^
43	GH20_15E	304.67 ± 4.53^k–m^	125.2 ± 1.74^d–p^	90.55 ± 0.91^o–v^	16.27 ± 0.07^i–n^	7.59 ± 0.18^m–p^	4.64 ± 0.04^j–q^	2.95 ± 0.17^s–w^
44	GH20_18B	318.83 ± 4.8^e–j^	138 ± 3.22^b–h^	90.63 ± 0.57^n–v^	12.98 ± 0.46^r–t^	7.73 ± 0.12^l–o^	4.41 ± 0.06^q–v^	3.32 ± 0.16^k–q^
45	GH20_16D	261.5 ± 5.51^s–u^	140.68 ± 2.61^a–d^	93.35 ± 0.89^b–e^	22.46 ± 0.34^a^	7.55 ± 0.17^n–p^	4.62 ± 0.06^j–q^	2.93 ± 0.11^s–w^
46	GH20_17B	332.3 ± 2.21^b–d^	137.33 ± 3.98^b–i^	90.55 ± 0.76^o–v^	16.25 ± 0.36^j–n^	8.60 ± 0.09^d–g^	5.36 ± 0.03^a–d^	3.24 ± 0.11^n–s^
47	GH20_17E	292.17 ± 2.91^n–p^	118.53 ± 1.75^k–q^	91.45 ± 0.79^i–q^	16.35 ± 0.2^i–n^	8.43 ± 0.06^e–i^	5.33 ± 0.08^a–d^	3.1 ± 0.08^o–u^
48	GH20_5B	259.13 ± 5.33^s–u^	124.73 ± 3.64^d–p^	91.54 ± 0.87^h–r^	16.55 ± 0.38^i–n^	8.71 ± 0.02^c–f^	4.74 ± 0.04^i–m^	3.97 ± 0.06^b^
49	GH20_11A	339.97 ± 0.84^ab^	133.93 ± 4.35^c–m^	94.3 ± 0.32^ab^	20.21 ± 0.7^a–e^	9.14 ± 0.04^ab^	5.27 ± 0.06^b–e^	3.86 ± 0.08^b–d^
50	GH20_12C	237.17 ± 6.21^vw^	120.93 ± 5.47^i–q^	90.37 ± 1.13^p–v^	12.14 ± 1.02^s–u^	8.42 ± 0.2^e–i^	4.79 ± 0.1^i–k^	3.64 ± 0.13^c–j^
51	GH20_18A	326.67 ± 5.65^c–f^	129.93 ± 3.62^c–p^	91.84 ± 0.71^e–p^	14.68 ± 0.96^n–r^	6.39 ± 0.16^t^	3.79 ± 0.05^w^	2.61 ± 0.2^xy^
52	GH20_20B	260.07 ± 2.03^s–u^	135.17 ± 6.39^c–j^	90.24 ± 0.53^q–v^	13.3 ± 0.51^q–s^	7.4 ± 0.05^o–q^	4.42 ± 0.05^p–v^	2.98 ± 0.08^r–v^
	Range	218.50–348.71	65.34–154.53	86.36–94.96	8.92–22.46	6.39–9.61	3.79–5.56	2.51–4.19
	Mean	297.60	124.69	91.86	17.00	8.12	4.76	3.36
	LSD (*p* ≤ 0.05)	12.04	16.49	1.56	2.27	0.39	0.25	0.30
	CV (%)	10.78	15.18	1.66	18.25	8.21	8.63	11.94

Values are mean ± SE. Means followed by different superscript letters within a column represent sampling significant differences.

The total carotenoids content was recorded as highest in the parental genotype Arka Kiran (9.55 mg/100 g), closely followed by the hybrid GH20_5A (9.13 mg/100 g), while it was lowest in Allahabad Safeda (0.78 mg/100 g). The highest lycopene content was found in GH20_5A (9.03 mg/100 g), which is comparable to Arka Kiran (8.7 mg/100 g), while lowest in hybrid GH20_5C (0.53 mg/100 g). Substantial variations were observed for total phenolic contents (TPC), total flavonoids content (TFC), and antioxidants activities (DPPH and FRAP assays) among the hybrids and their parents, indicating genetic influence on segregation patterns of fruit biochemical parameters (Table 5). The total phenolics content ranged from 218.50 to 348.71 mg GAE/100 g in the studied hybrids and parental genotypes, Arka Kiran exhibited the highest phenolics concentration (348.71 mg GAE/100 g), followed by Allahabad Safeda (340.77 mg GAE/100 g) while, GH20_12E recorded the lowest (218.50 mg GAE/100 g). The total flavonoids content varied from 65.34 to 154.53 mg QE/100 g. The highest flavonoids content was recorded in the Allahabad Safeda (154.53 mg QE/100 g), followed by the hybrid progeny GH20_8D (153.50 mg QE/100 g), while the lowest was in GH20_9A (65.34 mg QE/100 g). Total antioxidant activity measured by the DPPH assay also showed marked differences, ranging from 86.36 to 94.96% among the hybrids and parents. The parental genotype Arka Kiran demonstrated the highest DPPH activity (94.96%), and the lowest in GH20_12E (86.36%). Similarly, the FRAP assay revealed values ranging from 8.92 to 22.03 µmol Trolox/g amongst the hybrids and parents. The highest FRAP activity was observed in hybrids GH20_3A (22.01 µmol Trolox/g) and GH20_3D (22.03 µmol Trolox/g), both exceeding the values recorded in the parental genotypes Arka Kiran (20.69 µmol Trolox/g) and Allahabad Safeda (15.62 µmol Trolox/g).

### Multi-trait genotype-ideotype distance index (MGIDI) analysis

The multi-trait genotype-ideotype distance index (MGIDI) was employed to select the desirable guava hybrids among the studied progenies for an evidence-based multiple traits selection strategy. An ideotype was defined using key fruit quality and nutraceutical traits, including fruit weight, pulp thickness, TSS, titratable acidity, TSS/TA ratio, ascorbic acid, lycopene, total carotenoids, total phenolics, total flavonoids, DPPH, and FRAP. All traits were assigned positive directionality (higher values desirable) to reflect breeding objectives for improved fruit size, sweetness, and antioxidant potential. These parameters were selected as core attributes for new cultivar development, and genotypes were ranked based on their proximity to the predefined ideotype using the MGIDI index.

**Fig. 4:**
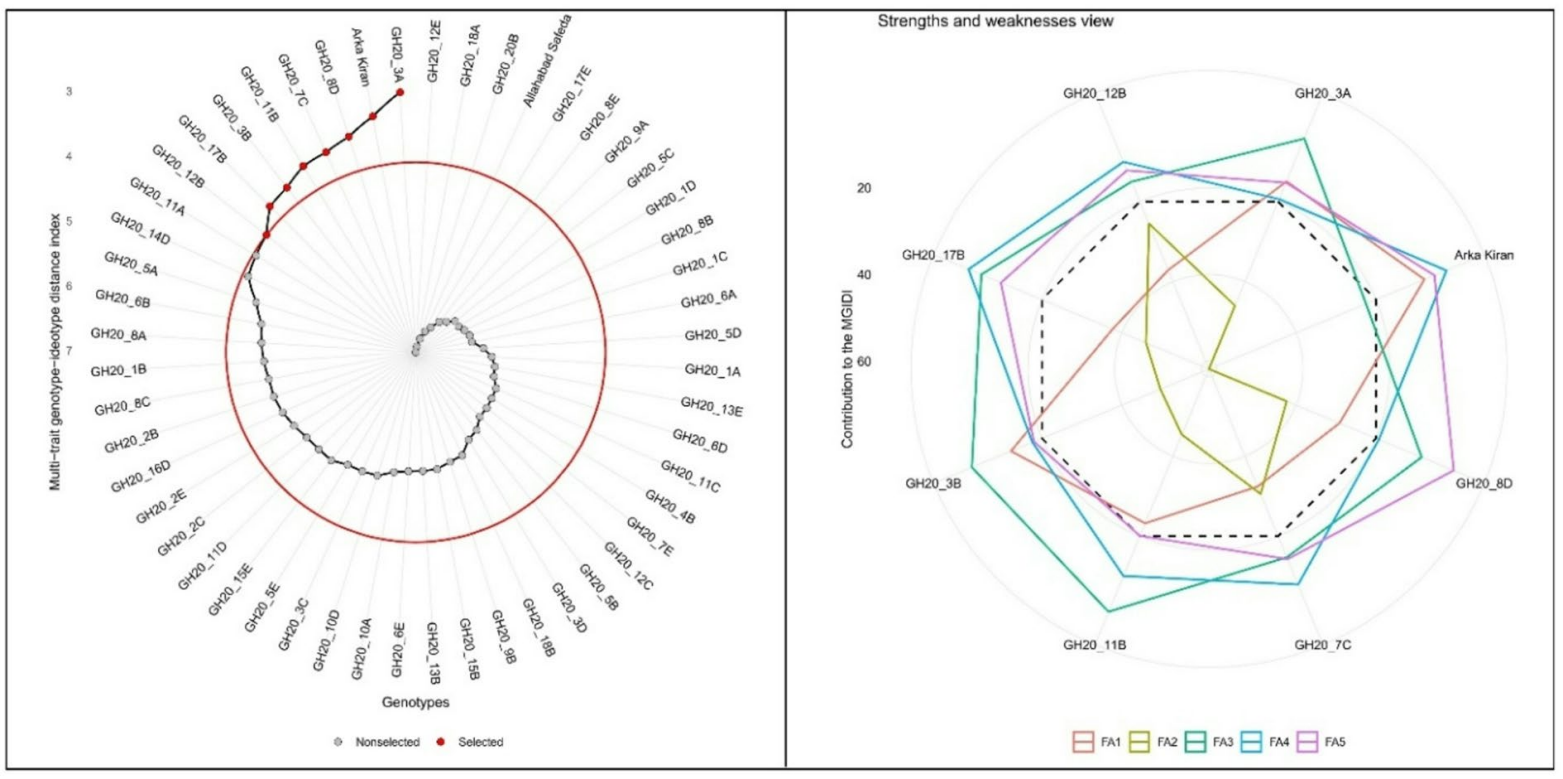
Multi-trait genotype–ideotype distance index (MGIDI) and strengths–weaknesses view illustrating selection performance across genotypes.

A total of eight genotypes, namely, GH20_3A, Arka Kiran, GH20_8D, GH20_7C, GH20_11B, GH20_3B, GH20_17B, and GH20_12B, were selected based on closeness to the ideotype. Although parental genotype Arka Kiran ranked favorably in the MGIDI analysis, the primary objective of the study was to identify superior full-sib hybrids. Therefore, emphasis was placed on selecting new hybrid progenies. The genotype GH20_3A had the lowest MGIDI score (3.01), followed by GH20_8D (3.54) and GH20_7C (3.64), indicating their superior overall performance across the studied fruit physical and biochemical parameters. These genotypes exhibited desirable combinations of morpho-biochemical characteristics like higher fruit weight, thicker pulp, elevated lycopene and total carotenoids content with enhanced antioxidants activities. However, genotype-specific variations (strength and weakness) were noted along the five significant dimensions having eigenvalue > 1.0 and contributing a cumulative 77.40% of variations (Supplementary Table 3 & Fig. 4). Although genotype GH20_11A recorded high fruit weight (406 g) and ascorbic acid content (302 mg/100 g), was not selected by the MGIDI index owing to its comparatively weaker performance in other traits such as lycopene and total carotenoids. The MGIDI index prioritizes balanced multi-trait performance rather than extreme superiority in any single trait.

### Pearson’s correlation studies

**Fig. 5:**
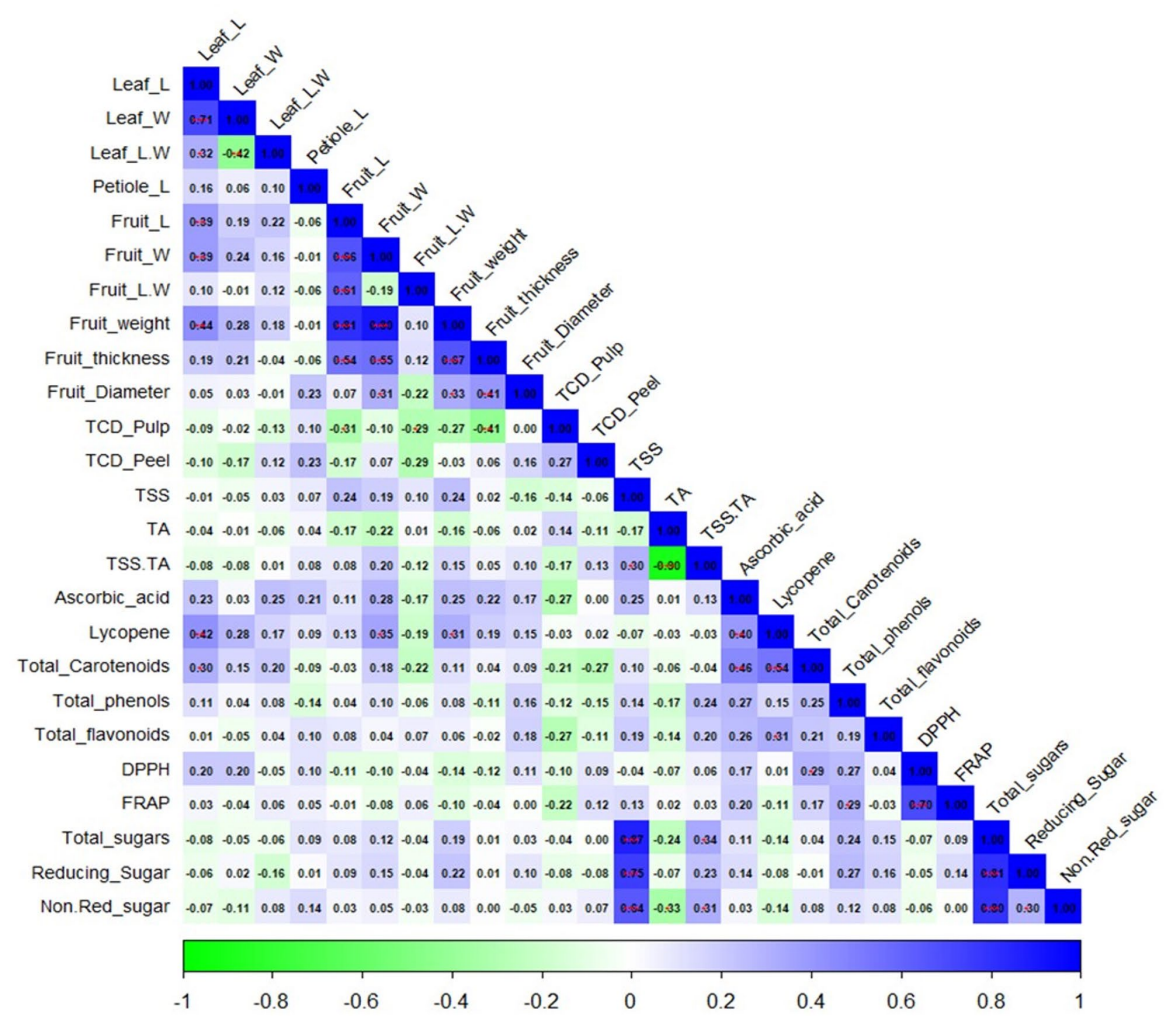
Simple correlations among quantitative parameters evaluated among the hybrid progenies and their parents. Stronger positive correlations are shown in deeper blue, while negative correlations are indicated by green.

The correlation analysis revealed clear patterns of linkage among studied morphological and biochemical parameters in guava hybrid progenies and parental genotypes (Fig. 5). Leaf length was positively correlated with leaf width (r = 0.74). Similarly, fruit size parameters (fruit length, width and weight) also showed significant correlation. Among the biochemical parameters, total soluble solids (TSS) showed a strong positive correlation with the TSS/TA ratio (r = 0.90) but a negative correlation with titratable acidity (r = –0.90). Further, total sugars were positively correlated with reducing sugars (r = 0.93), whereas non-reducing sugars showed negative correlations with total phenolics (r = –0.43), and TSS (r = − 0.44). The lycopene content was positively correlated with total carotenoids content (r = 0.54), and total phenolics content showed strong positive correlations with antioxidants activities (FRAP and DPPH assays). These two antioxidant assays were also positively correlated (r = 0.73), indicating consistency in oxidative scavenging assessment in guava.

### Principal component analysis (PCA)

Principal component analysis (PCA) was performed using quantitative traits to understand their contribution to morpho-biochemical diversity among the guava hybrids. A total of nine components were significant, with eigenvalues ≥ 1.0, and collectively explained 80.55% of total variation (Supplementary Table 4). PC1 explained 18.93% of the variability and was predominantly associated with fruit size parameters. High positive loadings were observed for fruit weight (0.90), fruit width (0.83), fruit length (0.73), pulp thickness (0.70), and leaf length (0.52), indicating that PC1 represents variation in overall fruit size and yield contributing attributes. PC2 contributed 13.81% (Supplementary Fig. 2) of the variation and was characterized by quality-related parameters with strong positive loadings for total sugars (0.71), TSS (0.67), reducing sugars (0.63), and non-reducing sugars (0.54). The PC3 explained 9.96% of the total variation and contributed mainly to variation in biochemical composition. Total carotenoids (0.60), lycopene (0.47), total phenols (0.46), DPPH activity (0.45), and FRAP activity (0.45) were positively associated with dimension, suggesting that these components capture the contribution of pigments and antioxidants capacity. PC4 (8.85%) was associated with ascorbic acid (0.40), TCD peel (0.42), and TCD pulp (0.45). PC5 (7.42%) showed strong positive loadings for titratable acidity (0.66) and fruit length–width ratio (0.49), and a negative loading for TSS/TA (–0.70), collectively reflecting the acidity–sweetness balance.

**Fig. 6:**
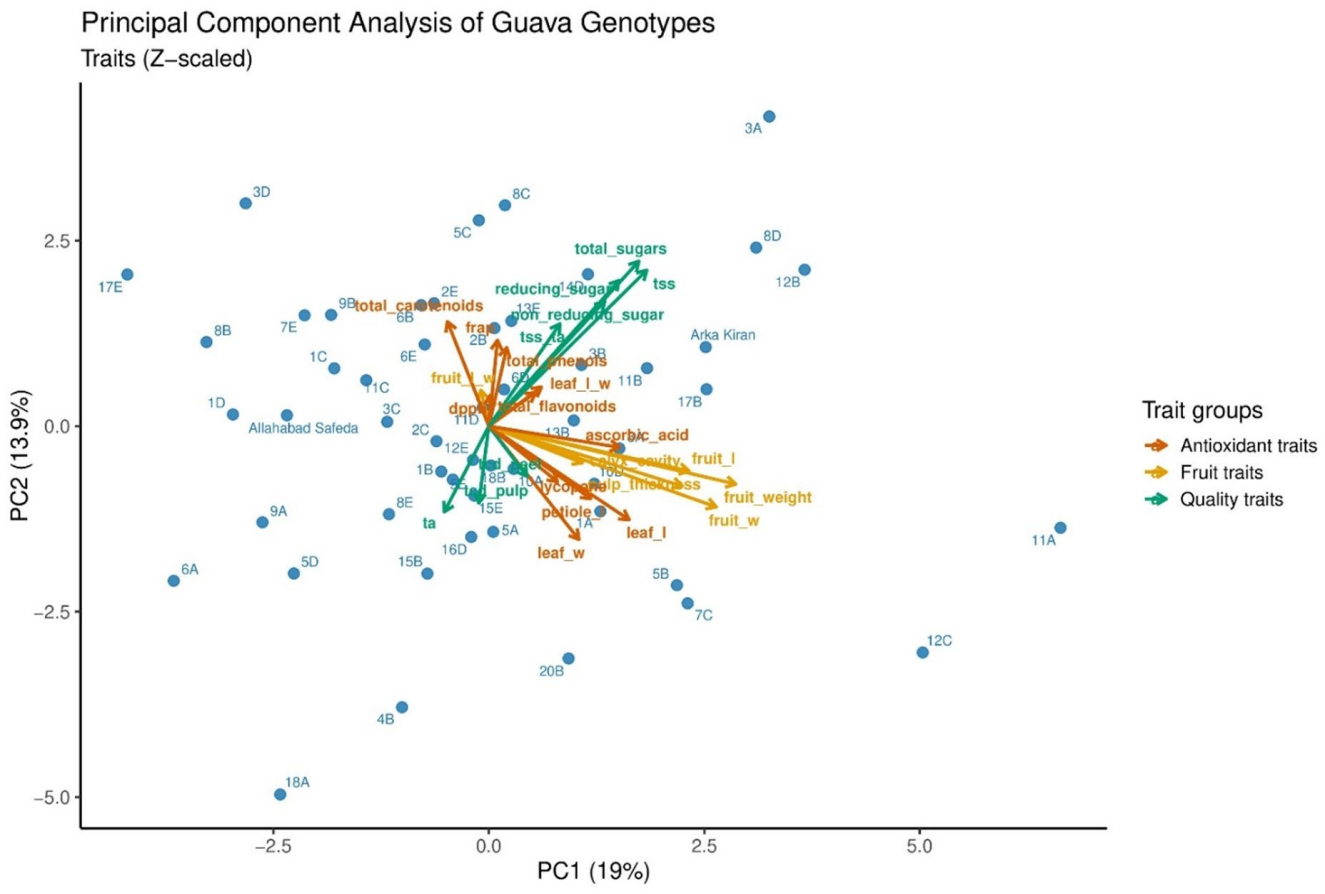
PCA bi-plots based on evaluated morpho-biochemical parameters among the hybrids and their parents. Genotypes are represented by labels and trait vectors indicate the direction and magnitude of trait contributions. [All genotypes’ names in this figure are preceded by GH20_].

In the PCA biplot, the first two principal components explained 32.9% of the total variation, with PC1 and PC2 accounting for 19.0 and 13.9%, respectively (Fig. 6). PC1 distinctly separated genotypes based on fruit size and quality attributes, in contrast to antioxidant parameters. Positive loadings on PC1 were mainly influenced by fruit weight, fruit length, fruit width, ascorbic acid, TSS, and total sugars, indicating that genotypes positioned on the positive side of this axis possess superior fruit size and quality parameters. Conversely, negative PC1 loadings were mainly associated with total carotenoids, FRAP and DPPH activities, reflecting a higher antioxidant potential. PC2 further discriminated genotypes based on sugars and leaf parameters, with TSS, total sugars, reducing sugars, and non-reducing sugars contributed positively, whereas leaf length, leaf width and pulp thickness showed negative loadings. Genotypes GH20_3A, GH20_8D, GH20_12B, Arka Kiran, and GH20_11B, clustered in the positive PC1 and positive PC2 quadrant, exhibited strong associations with fruit quality and sweetness. In contrast, genotypes GH20_3D, GH20_2E, GH20_17E, and GH20_7E, grouped in the negative PC1 and positive PC2 quadrant, were associated with antioxidant-related parameters, highlighting their potential nutritional significance. Genotypes positioned in the negative PC1 and PC2 quadrant (GH20_18A, GH20_4B, GH20_6A, and GH20_5D) showed stronger associations with acidity and comparatively lower fruit quality attributes. Meanwhile, genotypes like GH20_7C, GH20_5B, GH20_11A, and GH20_12C, grouped in the positive PC1 and negative PC2 quadrant, were characterized by larger fruit size with moderate quality parameters. The clear separation of fruit size and antioxidant-related traits across different quadrants offers an opportunity for selecting guava genotypes aligned with breeding goals or consumer preferences. The parental genotypes Allahabad Safeda and Arka Kiran occupied distinct positions along the principal components, reflecting contrasting contributions of fruit quality and antioxidant traits to their hybrid progenies.

### Hierarchical clustering

**Fig. 7:**
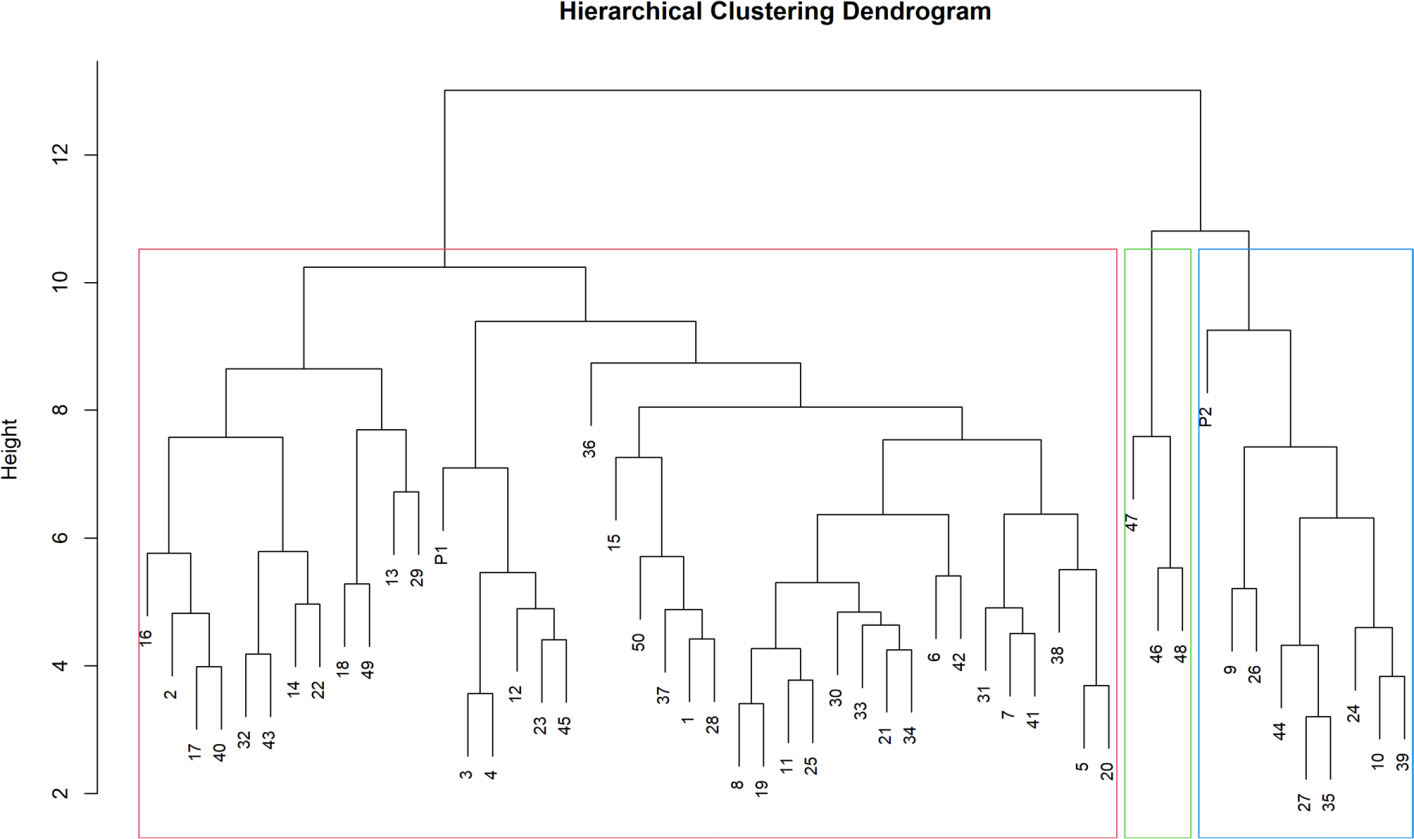
Clustering patterns of hybrid progenies and their parental genotypes based on morpho-biochemical traits.

Hierarchical clustering analysis of 50 guava hybrids and their two parental genotypes based on morpho-biochemical traits grouped them into three major clusters (Fig. 7), clearly demonstrating substantial genetic variability within the hybrid population. Cluster I was the largest and was further sub-divided into two clades, indicating a high degree of similarity of guava hybrids within the cluster/clade. In this cluster, the female parent, Allahabad Safeda (P1), grouped closely with a large proportion of hybrid progenies, suggesting that many full-sib hybrids share a greater phenotypic resemblance to P1 for the evaluated traits. Cluster II consisted of only three distinct genotypes, namely, GH20_5B, GH20_2A, and GH20_11A, which were moderately divergent from the main group, indicating the presence of rare recombinants with distinct trait expressions. Cluster III comprised eight genotypes, including the male parent Arka Kiran (P2), which formed a distinctly separated group, highlighting its unique morpho-biochemical profile and close resemblance to the members within the cluster.

## Discussion

Confirmation of hybridity is a prerequisite in perennial fruit breeding to ensure the authenticity of progenies derived from controlled crosses of desirable parents. In the present study, three polymorphic g-SSR markers (FHTGSSR-3.4, FHTGSSR-7.5, and FHTGSSR-3.6) reliably discriminated guava parental genotypes and validated the hybrid status of derived progenies. The successful amplification of both parental alleles in confirmed hybrids demonstrates the utility of SSR markers as robust tools for early-stage hybrid authentication in guava breeding programs, consistent with previous reports emphasizing their co-dominant inheritance and reproducibility^21–23^.

Morphological characterization is essential for taxonomic studies and the classification of genotypes in fruit crop^16^. The wide morphological variability among the hybrid progenies explicitly shows the effectiveness of hybridization between heterozygous parents in perennial fruit species like guava. Despite being evaluated for the first time, these hybrids showed morphological variability comparable to that reported in guava and related *Psidium* genotypes^24^. Due to their genetic basis and ease of field observation, these morphological features offer an effective means of distinguishing individual hybrid progeny^25^. The results showed that morphological variation in hybrid genotypes exceeded that of parental traits, indicating transgressive segregation. Tree architecture is an essential trait for selection, as spreading and genetically dwarf growth habits are desirable for high-density planting^26^. In this study, clear segregation in branch orientation was observed, with 21 hybrids showing predominance of spreading habit, unlike the erect and drooping patterns of the parents. These findings are in line with previous studies on guava populations^27,28^. Furthermore, variations in leaf morphology and leaf size in the guava hybrid population agree with previous findings by Ran et al.^29^, Vishwakarma et al.^23^ and Nagar et al.^28^. Overall, these results confirm substantial genetic variability in leaf traits, providing practical approach for genotype characterization and selection. Differences in leaf size and shape can serve as rapid, cost-effective indices for early-stage screening of the hybrids in guava breeding program.

Similarly, fruit morphological variation was also evident among the newly developed guava hybrids, consistent with earlier reports in guava^24,27,28,30^. Some of the studied traits were having extreme values of both the parent, indicating their transgressive segregation. The red pulp trait in guava, highly valued for its consumer appeal and enriched with lycopene and β-carotene, was expressed in 90% of the studied hybrid progenies, demonstrating strong dominance inherited from the male parent, Arka Kiran. The presence of white pulp in only five progenies suggests recessive control of the white pulp phenotype over red/pink pulp colour. Furthermore, the findings confirm that white pulp colour is governed by a homozygous recessive trait, whereas guava parent heterozygous for pink/red pulp colour genes produce segregating progenies, consistent with the earlier report of Thakre et al.^31^. Significant variability was observed for fruit shape, size, weight, and pulp thickness, which are the key criteria for genotype selection in both fresh and processing purposes. The variation in fruit physical parameters observed amongst the guava hybrids aligns with previous findings^32–34^. Fruit weight exhibited nearly five-fold variation (79.33–406.33 g), with several hybrids even surpassing from commercial cultivars like Allahabad Safeda, L-49 (200 g), and Shweta (359 g)^24^, highlighting substantial breeding potential. The pulp thickness ranged from 1.27 to 2.46 cm, and superior genotypes such as GH20_11A, with thicker pulp, are promising for commercial evaluation and fruit size improvement.

Furthermore, newly developed guava hybrids exhibited substantial variations for the assayed biochemical parameters, viz., TSS, acidity, sugars, total carotenoids, lycopene, total flavonoids, and antioxidants activities. This variability reflects parental heterozygosity and recombination, generating novel phenotypes with strong potential for commercial selection and breeding. The observed ranges of biochemicals and antioxidants recorded in the present study were consistent with previous reports in guava^5,33,35,36^. The fruit pulp, along with TSS and sugar contents are key determinants imparting sweetness and consumer acceptance in guava. In this study, several hybrids outperformed both parent genotypes, Allahabad Safeda and Arka Kiran, in terms of TSS, with GH20_3A and GH20_12B recording values above 13.5°Brix. Hybrids with lower acidity, such as GH20_5C, showed high TSS/acid ratios, indicating enhanced sweetness. Similar to earlier findings in guava, sweetness variation is primarily governed by genotypic differences and enzymatic regulation of sucrose conversion into reducing sugars during fruit maturation^37^. Segregation for sugar–acid balance was evident, and genotypes combining high TSS and total sugars, like GH20_3A and GH20_8D, are promising candidates for fresh consumption. Ascorbic acid content varied widely among the hybrids, with GH20_11A showing exceptionally high vitamin C (302.20 mg/100 g), surpassing both parents, consistent with the quantitative inheritance of this trait in guava. Total carotenoids and lycopene contents also showed marked variability, particularly among red-pulped hybrids, with GH20_5A and GH20_17B exhibiting lycopene levels comparable to Arka Kiran, confirming effective introgression of pigment-related genes.

Phenolics, flavonoids and antioxidants activity also varied substantially across hybrids, highlighting their nutraceutical potential. Although the parental genotypes generally exhibited higher levels of phenolics and flavonoids, hybrids such as GH20_8D and GH20_3A demonstrated antioxidant activities that were comparable to, or even surpassed, those of the parents. Superior FRAP activity in GH20_3A and GH20_3D suggests positive transgressive segregation, indicating that antioxidant potential results from synergistic interactions among vitamin C, total carotenoids, and other bioactive compounds rather than phenolic content alone^38^. Independent segregation of sugars, acidity, vitamin C, total carotenoids, and antioxidant activity enables targeted selection for fresh consumption and processing. Several genotypes (GH20_3A, GH20_5C, GH20_11A, GH20_5A, GH20_3D, and GH20_8D) demonstrated desirable quality attributes with enhanced antioxidant potential.

Multi-trait evaluation using the MGIDI index further validated these findings by integrating correlated traits into a single selection criterion, providing a comprehensive assessment of genotypic performance and facilitating the identification of superior genotypes closest to the predefined ideotype^39,40^. A total of seven new guava hybrids (GH20_3A, GH20_8D, GH20_7C, GH20_11B, GH20_3B, GH20_17B, and GH20_12B) exhibited superior proximity to the ideotype, reflecting their desirable multi-trait performance. The strength–weakness view revealed that trait contributions were distributed across five significant dimensions (eigenvalues > 1), explaining 77.4% of the total variation, confirming that the observed superiority was not driven by a single trait but by a balanced contribution from multiple fruit and nutraceutical attributes. The identification of hybrids combining yield potential with nutraceutical quality underscores the effectiveness of MGIDI in perennial fruit breeding. Notably, GH20_3A, GH20_8D, and GH20_7C emerged as promising candidates for commercial evaluation and future hybridization, while GH20_11A represents a valuable donor for fruit weight and vitamin C enhancement.

Multivariate analyses (PCA and hierarchical clustering) effectively characterized the population, with nine principal components (eigenvalues ≥ 1) explaining 80.55% of total variation. This confirms that the measured traits adequately captured phenotypic diversity. The distribution of variance across multiple components reflects parental heterozygosity, while PCA reduced data complexity and highlighted key trait associations for efficient genotype evaluation. PCA biplot analysis was used to examine genotype distribution and trait associations, with the first two components explaining 32.9% of total variation and effectively capturing genotype parameter relationships. This is consistent with previous guava studies reporting 28.99% variation across 18 genotypes^35^, 74.5% variation across 12 genotypes^41^, and 37.3% across 51 genotypes^42^. Fruit related parameters clustered tightly, indicating strong positive correlations similar to those reported by Kumari et al.^30^, whereas antioxidant parameters (DPPH, FRAP, and total carotenoids) formed distinct groups, reflecting their independent contribution to variability. Genotypes distributed across different quadrants represent valuable resources for breeding programs aiming to combine productivity, fruit quality, and nutritional attributes.

Hierarchical clustering results were consistent with correlation analysis, revealing strong interrelationships among fruit biochemical parameters and plant traits. These findings indicate the feasibility of simultaneous improvement of multiple quality attributes, as previously reported by Singh et al.^43^, Kumari et al.^30^ and Gangappa et al.^24^. The 52 guava genotypes including hybrids and parents were grouped into three major clusters, supporting PCA outcomes. The largest cluster included Allahabad Safeda and several phenotypically similar hybrids, suggesting directional inheritance of fruit size related traits. A small, distinct Cluster II comprising only three hybrids indicated recombination-driven rare variants, while Cluster III grouped Arka Kiran with 8 hybrids characterized by higher pigments and antioxidants accumulation, reflecting inheritance from the male parent. The hybridization between genotypes from genetically distinct clusters enhances the potential for combining desirable traits and generating transgressive segregants with superior fruit quality. Multivariate analyses confirmed substantial phenotypic and biochemical diversity within the hybrid population, providing a strong genetic base for selection^35,41–45^. The clear differentiation between high-yielding and bioactive-rich genotypes indicates that simultaneous improvement in fruit size and nutraceutical attributes is feasible. These findings also demonstrate the effectiveness of PCA and cluster analysis for early-generation hybrid evaluation and strategic breeding decisions in guava improvement programs.

## Conclusion

This study revealed considerable morphological and biochemical diversity among guava hybrids developed from the parental genotypes ‘Allahabad Safeda’ and ‘Arka Kiran’. Hybridity confirmation through SSR markers supported the genetic authenticity of the evaluated progenies, while significant variation in fruit size, pulp thickness, sweetness, vitamin C, total carotenoids, and antioxidant activities highlighted the effectiveness of hybridization of two distinct heterozygous guava parents for the development of segregating progenies and identification of novel recombinants as an elite genotype or unique genetic resources. Further, multivariate analyses, particularly the MGIDI index, proved effective in integrating multiple correlated traits into a single selection framework. Hybrids such as GH20_3A, GH20_8D, and GH20_7C ranked highest among the seven MGIDI-selected genotypes and showed superior proximity to the ideotype, reflecting balanced performance across fruit quality and bioactive attributes. Genotypes with high TSS and a favourable sugar–acid balance, such as GH20_3A and GH20_8D, are promising for fresh consumption, whereas those enriched in carotenoids, vitamin C, and antioxidant activity hold potential for processing and nutraceutical-oriented breeding programs. The identification of unique hybrid, GH20_11A, combining superior fruit size with enhanced nutraceutical potential, demonstrates the feasibility of simultaneous improvement of yield-related and functional quality traits in guava. However, as the hybrids were evaluated under a single environmental condition, multi-location and multi-season replicated clonal trials are essential to confirm trait stability and performance across diverse environments before commercial release. Overall, the integration of SSR-based hybrid validation, morphological and biochemical characterization, and multi-trait selection has enabled the identification of elite hybrid candidates, laying a strong foundation for targeted genetic improvement and future varietal development in guava.

## Materials and methods

### Plant materials

In this study, four-year-old 60 hybrid progenies derived from the cross between ‘Allahabad Safeda’ (♀) and ‘Arka Kiran’ (♂) were initially screened for hybridity using SSR markers. Of these, 56 were validated as true hybrids, and 50 progenies that fruited during the Mrig bahar season of 2024, along with their parental genotypes, were selected for multivariate analyses (Fig. 2). In this investigation, all morphological and biochemical parameters were recorded from sub-samples within individual trees rather than from independent experimental replications. The guava parental genotypes undertaken for hybridization selected from the field gene bank of the Division of Fruits and Horticultural Technology (FHT), ICAR-Indian Agricultural Research Institute (IARI), New Delhi, with authorization from the institute. The hybridization between selected guava parents was carried out during the flowering month (April) of the year 2020 using the conventional method of emasculation followed by controlled pollination. The taxonomic identity of parents and their progenies was confirmed using morphological descriptors and authenticated reference materials. A representative voucher specimen of the germplasm has been deposited in the herbarium of ICAR-IARI, New Delhi, for future reference. Ripened fruits collected from tagged branches were utilized for seed extraction and subsequent development of hybrid progenies. These newly developed hybrids were established and planted at 3 m × 4 m spacing at the experimental orchard of the Division of FHT, ICAR-IARI, New Delhi (28°40′N, 77°12′E; 228.60 m above the mean sea level). The climatic conditions of the experimental site were typical of a sub-tropical climate. The weather conditions recorded in the experimental orchard during the study are provided in Supplementary Table 5. The studied guava hybrids and their parental genotypes were subjected to uniform management practices as recommended for the crop.

### Ascertaining hybridity

Genomic DNA was isolated from young leaves of guava hybrid progenies and parents using a modified CTAB protocol^46^. DNA concentration and purity were determined using a NanoDrop spectrophotometer (Thermo Fisher Scientific, USA), and integrity was confirmed by electrophoresis on a 0.8% agarose gel. A total of 46 genome-wide SSRs were chosen for ascertaining the hybridity^47^. The primers were synthesized (GeneDireX, Inc.), reconstituted in TE buffer to obtain 100 pmol/µL stock and 10 pmol/µL working solutions, which were then stored at –20 °C. The PCR conditions for markers were optimized using gradient PCR.

The PCR amplification was performed, and amplified PCR products, along with a DNA ladder (DNAmark™ 100 bp), were loaded onto a 2.5% agarose gel containing ethidium bromide (18 μl per 500 mL of gel) in 1X TAE buffer. Electrophoresis was conducted at a constant voltage of 5 V/cm for approximately 2.5 h. The DNA bands were then visualized under UV light and recorded using a gel documentation system (Gel Luminax, Zenith). The SSR amplicons were evaluated for reproducibility, clarity, and polymorphism between parental genotypes, and were subsequently used to assess hybridity among the developed hybrid progenies.

### Morphological characterization

Plant morphological parameters of the studied hybrid population and parents were carried out following the Guava Descriptor developed by the Protection of Plant Varieties and Farmers’ Rights Authority^17^, Government of India, New Delhi, and the International Union for the Protection of New Varieties of Plants (UPOV, 1987)^48^. The category mentioned in the descriptor was noted for each trait after observing the plant and its leaves. For the assessment of leaf parameters, fifteen fully mature leaves were selected and observations were recorded. Leaf dimensions, such as length and width, were measured using digital Vernier calipers (Mitutoyo Inc., Japan).

### Fruit physical parameters

From each hybrid tree, fifteen fruits were randomly harvested at physiological maturity to obtain average fruit phenotypic values. Fruit size parameters, such as fruit length and width, were measured using digital Vernier calipers (Mitutoyo Inc., Japan). Fruit weight was measured using a digital weighing balance (Citizen Scale Pvt. Ltd, India). The fruit was cut at the equatorial plane, and the pulp thickness was measured in millimeter (mm) using digital Vernier calipers.

Fruit peel and pulp colour were estimated using the RHS colour chart and further quantified using a Hunter Lab colorimeter (Labscan XE) in Hunter coordinates: ‘L*’ (lightness), ‘a*’ (redness and greenness) and ‘b*’ (yellowness and blueness)^49^.

TCD (Total colour difference) was calculated using the following equation:

$$TCD = \left[ {\left( {\Delta L^*} \right)2 + \left( {\Delta a^*} \right)2 + \left( {\Delta b^*} \right)2} \right]1/2$$

where, ΔL^*^, Δa^*^ and Δb^*^ represent the difference in L^*^, a^*^ and b^*^ values at a particular interval from the respective initial values.

### Pulp biochemical parameters

The biochemical parameters, namely total soluble solids (TSS), titratable acidity (TA), TSS/TA ratio, and sugars content, were estimated in triplicates from the fresh fruit pulp obtained from hybrid progenies and their parental genotypes.

The total soluble solids (TSS) of fresh fruit pulp were quantified with the help of a digital refractometer (MA871; Milwaukee, Romania). A drop of guava juice from each sample was placed on the prism surface, and values were recorded in °Brix. The refractometer was standardized with a known sugar concentration before taking the observations. The titratable acidity (TA), was estimated using acid–base titration as described by the Association of Official Agricultural Chemists^50^. Briefly, 2 g of fruit pulp was crushed with double distilled water, and the final volume was adjusted to 20 mL. The mixture was then filtered through Whatman filter paper. A 10 mL aliquot of filtrate was titrated against 0.1 N NaOH using phenolphthalein as an indicator until a stable pale-pink endpoint appeared. TA was calculated as a percentage of citric acid equivalents. The TSS/TA ratio was also calculated from the estimated TSS and TA for each fruit sample.

Total sugars and reducing sugars in guava pulp were quantified using the Lane and Eynon titrimetric method, which employed Fehling’s solutions A and B^51^. Fresh pulp (25 g) was homogenized with double distilled water and diluted to 100 mL. The clarification was achieved by adding 2 mL of lead acetate, followed by 2 mL of potassium oxalate to precipitate the excess lead. The volume was adjusted to 250 mL using distilled water and filtered to obtain a clear extract for titration. For total sugars, 25 mL of the filtrate was hydrolyzed with 5 mL concentrated HCl and boiled for 30 min to convert non-reducing sugars into reducing sugars. The mixture was neutralized with NaOH using phenolphthalein as an indicator. Equal volumes (5 mL each) of Fehling’s solutions A and B were mixed in a conical flask, diluted, and heated to boiling. The prepared extract was titrated against the boiling Fehling’s mixture in the presence of methylene blue until the disappearance of blue colour indicated the endpoint. The Fehling’s factor was determined using a standard glucose solution.

The content of total sugars was calculated using the following formula:

$$Total\;sugars \left( \% \right) = \frac{Fehling^{\prime}s factor}{{Titre\;value}} \times \frac{{Final\;Volume \left( {mL} \right) }}{Sample\;Weight \left( g \right)} \times 100$$

where,

$$Fehling^{\prime}s\;factor = \frac{Titre \times 25}{{1000}}$$

For reducing sugars, a direct titration of the clarified sample extract (without hydrolysis) was performed using Fehling’s reagents under identical conditions, and the calculation was based on the same formula used for total sugar content.

The content of non-reducing sugars was estimated using the following formula:

$$\mathrm{Non}-\text{reducing sugar }\left(\mathrm{\%}\right)=\text{Total Sugars}-\text{ Reducing sugars }\times 0.95$$

### Ascorbic acid content

Ascorbic acid content in guava pulp was quantified using the standard AOAC titrimetric method^52^. Fresh fruit pulp (2 g) was homogenized with 20 mL of 3% metaphosphoric acid and filtered through Whatman No. 1 filter paper to obtain a clear extract. A 5 mL aliquot of the extract was titrated against standardized 2,6-dichlorophenol-indophenol (DCPIP) dye until a light pink endpoint persisted for 15 sec. The DCPIP dye was standardized using ascorbic acid prepared in 3% metaphosphoric acid, and the dye factor (DF) was calculated as:

$$\text{Dye Factor }(\mathrm{DF})=\frac{0.5}{\mathrm{Titre}}$$

Ascorbic acid content in fresh fruit pulp was calculated using the following formula.

$$Ascorbic\;acid \left( {mg/100 \; FW} \right) = \left( {DF \times Titre\;value} \right) \times \frac{Volume\;made (mL)}{{A\;liquot\;volume (mL) \times Sample\;weight (g)}} \times 100$$

### Total phenolics content (TPC)

Total phenolics content was quantified using the Folin–Ciocalteu colourimetric method^53^. One gram of fresh pulp was homogenized in 80% ethanol, then centrifuged at 10,000 × g for 20 min. The supernatant was collected and stored at − 20 °C until analysis. For each assay, 100 µL of extract was mixed with 0.5 mL Folin–Ciocalteu reagent (0.2 M) and 2.9 mL double distilled water. After 10 min, 2 mL of a 20% (m/v) sodium carbonate solution was added, and the reaction mixture was incubated for 30 min at room temperature. Absorbance was recorded at 760 nm using a UV–Vis spectrophotometer (GENESYS; Thermo Fisher Scientific, USA). The TPC in fresh fruit pulp was quantified with the help of a gallic acid standard calibration curve, and the results were expressed as mg gallic acid equivalents per 100 g fresh weight (mg GAE 100 g^−1^ FW).

### Total flavonoids content

Total flavonoids content was estimated using the aluminium chloride colourimetric method described by Zhishen et al.^54^. An aliquot of 1 mL aliquot of the ethanolic extract (prepared as for TPC) was mixed with 1.4 mL distilled water and 0.3 mL of 5% (m/v) sodium nitrite solution. After 5 min, 0.3 mL of 10% (m/v) aluminium chloride was added, and the reaction was allowed to proceed for 6 min, followed by the addition of 2 mL of 1 M sodium hydroxide. The final volume was made up to 5 mL with distilled water, and the mixture was vortexed. Absorbance was recorded at 510 nm using a UV–Vis spectrophotometer (GENESYS; Thermo Fisher Scientific, USA). Quantification was performed with the help of a quercetin standard calibration curve and expressed as mg quercetin equivalents per 100 g sample (mg QE 100/g sample).

### Lycopene and total carotenoids content

The lycopene and total carotenoids content of guava pulp were quantified following the spectrophotometric method^55–58^. The fresh guava pulp (2 g) was homogenized in chilled acetone to facilitate complete pigment extraction, and the extraction was continued until the residue became colourless. The pooled acetone extract was transferred to a separating funnel containing petroleum ether, and the pigments were allowed to partition into the organic phase by gentle shaking and gradual addition of double distilled water. The aqueous layer was discarded, and the petroleum ether layer containing carotenoid pigments was collected in amber bottles to prevent photo-oxidation. Extraction was repeated with fresh petroleum ether until no further colour was observed in the solvent. Anhydrous sodium sulphate was added to remove residual moisture, and the extract was diluted to 50 mL.

The absorbance of the pigment extract was recorded using a UV–Vis spectrophotometer (GENESYS; Thermo Fisher Scientific, USA) at 452 nm for total carotenoids and 503 nm for lycopene, with petroleum ether used as the blank. Lycopene and total carotenoids content is calculated based on their respective extinction coefficients (β-carotene and lycopene in petroleum ether; used as the reference extinction coefficient for total carotenoids and lycopene in petroleum ether, respectively) and expressed as milligram per 100 g of fresh fruit weight.

### Total antioxidants bioassay

The total antioxidants activity of guava fruit pulp was evaluated using two complementary assays, i.e., DPPH (2, 2-diphenyl-2-picryl-hydrazyl) free-radical scavenging activity and ferric reducing antioxidant power (FRAP) assay.

The DPPH assay was performed following Brand-Williams et al.^59^ with minor modifications. The DPPH working solution (0.025 g/L) was prepared in 70% methanol (v/v) and stored in amber coloured glassware. The absorbance of the solution was adjusted to 0.70 ± 0.02 at 517 nm. Fruit extracts were prepared as described for TPC analysis. For each sample, 0.1 mL of extract was mixed with 2.9 mL of DPPH solution, vortexed, and incubated in the dark at room temperature for 30 min. Absorbance was recorded at 517 nm using a UV–Vis spectrophotometer (GENESYS; Thermo Fisher Scientific, USA). Antioxidant capacity was expressed as percentage radical scavenging activity.

Ferric reducing activity was determined using the FRAP method^60^ with some modifications. The FRAP reagent was freshly prepared by mixing acetate buffer (300 mM, pH 3.6), TPTZ solution (10 mM in 40 mM HCl), and FeCl_3_·6H_2_O (20 mM) in a 10:1:1 ratio. An aliquot of 0.1 mL of fruit extract was added to 3 mL of FRAP reagent, vortexed, and incubated in the dark for 30 min. The development of the blue ferrous–TPTZ complex was measured at 593 nm using a UV–Vis spectrophotometer. Antioxidant activity was calculated using a Trolox standard curve and expressed as micromoles Trolox equivalents per gram fresh weight (µM TE g^−1^ FW).

### Statistical analyses

The quantitative morphological and biochemical datasets were subjected to one-way analysis of variance (ANOVA) using SAS statistical software (version 9.3; SAS Institute Inc., Cary, NC, USA). Following a significant F-test, mean separation among genotypes was carried out using Fisher’s Least Significant Difference (LSD) test at p ≤ 0.05. Qualitative morphological traits were summarized using frequency distribution and bar charts generated in Microsoft Excel (version 12.0; Microsoft Corp., USA. To assess multivariate variation among hybrids and parents, principal component analysis (PCA), hierarchical cluster analysis (Euclidean distance with Ward’s method), Pearson’s correlation matrix, and MGIDI (Multi-trait genotype-ideotype distance index) were conducted using R Studio (Version 2022.07.1–554; R Core Team) employing the following R packages: *FactoMineR, factoextra, ggrepel, metan, ggplot2, and corrplot*.

## Supplementary Information

Below is the link to the electronic supplementary material.

Supplementary Material 1

Supplementary Material 2

## Data Availability

Data supporting this study’s findings can be obtained from the corresponding author upon reasonable request.
