# Supplementary material for: Multivariate assessment of newly developed guava (Psidium guajava L.) hybrids for tree and fruit quality traits
Source: Sci Rep. 2026 Apr 4;16:16246. doi: 10.1038/s41598-026-45320-8 (PMC13201807; doi:10.1038/s41598-026-45320-8)
Supplement: Supplementary file 1 — Supplementary Material 1 [file 41598_2026_45320_MOESM1_ESM.docx]

**S. Table 1.** Variation in the leaf qualitative morphological parameters among newly developed guava hybrids and parental genotypes.

| **S. No** | **Tree: attitude of branches** | **Bark colour** | **Young shoot: colour of stem** | **Leaf: shape** | **Leaf: shape of tip** | **Leaf: shape of base** | **Petiole orientation** | **Leaf: twisting** | **Young leaf: anthocyanin coloration** | **Leaf: variegation** | **Leaf: colour** | **Leaf: pubescence on lower side** | **Colour of lamina** | **Lamina thickness** | **Lamina pubescence** | **Colour of leaf during winter** |
| --- | --- | --- | --- | --- | --- | --- | --- | --- | --- | --- | --- | --- | --- | --- | --- | --- |
| **P_1_** | Spreading | Light green | Green | Obovate | Obtuse | Cordate | Straight | Absent | Absent | Absent | Green group | Sparse | Green | Intermediate | Sparse | Coppery |
| **P_2_** | Spreading | Whitish | Green | Oblanceolate | Acute | Rounded | Straight | Absent | Present | Absent | Green group | Sparse | Light green | Intermediate | Absent | Red |
| **1** | Spreading | Light green | Green with red streaks | Oblanceolate | Acute | Cordate | Twisted | Present | Present | Absent | Green group | Sparse | Green | Thin | Absent | Red |
| **2** | Drooping | Light green | Green with red streaks | Ovate | Acute | Rounded | Twisted | Absent | Present | Absent | Green group | Sparse | Dark green | Thin | Dense | Coppery |
| **3** | Erect | Creamy | Green with red streaks | Ovate | Obtuse | Cordate | Twisted | Absent | Present | Absent | Green group | Sparse | Dark green | Intermediate | Absent | Brick red |
| **4** | Spreading | Creamy | Green with red streaks | Oblong | Acute | Rounded | Straight | Present | Present | Absent | Green group | Sparse | Green | Thin | Very dense | Red |
| **5** | Erect | Light green | Green with red streaks | Oblong | Rounded | Cordate | Twisted | Present | Absent | Absent | Green group | Sparse | Light green | Thick | Sparse | Red |
| **6** | Erect | Light green | Green with red streaks | Obovate | Rounded | Cordate | Straight | Absent | Present | Absent | Green group | Sparse | Light green | Thick | Sparse | Brick red |
| **7** | Drooping | Whitish | Green with red streaks | Oblanceolate | Obtuse | Cordate | Straight | Absent | Absent | Absent | Green group | Sparse | Green | Intermediate | Dense | Coppery |
| **8** | Spreading | Whitish | Green with red streaks | Ovate | Obtuse | Cordate | Twisted | Present | Present | Absent | Green group | Sparse | Light green | Intermediate | Dense | Coppery |
| **9** | Spreading | Creamy | Green with red streaks | Oblong | Acute | Rounded | Twisted | Present | Present | Absent | Green group | Sparse | Green | Thin | Dense | Coppery |
| **10** | Spreading | Whitish | Green | Ovate | Obtuse | Rounded | Straight | Absent | Absent | Absent | Green group | Sparse | Dark green | Thin | Absent | Coppery |
| **11** | Drooping | Creamy | Green with red streaks | Oblong | Obtuse | Rounded | Twisted | Present | Absent | Absent | Green group | Sparse | Dark green | Intermediate | Sparse | Red |
| **12** | Drooping | Creamy | Green with red streaks | Ovate | Obtuse | Cordate | Straight | Present | Absent | Absent | Green group | Sparse | Green | Intermediate | Absent | Red |
| **13** | Drooping | Whitish | Green with red streaks | Ovate | Obtuse | Rounded | Twisted | Absent | Absent | Absent | Green group | Sparse | Green | Thick | Dense | Coppery |
| **14** | Erect | Whitish | Dark red | Ovate | Obtuse | Rounded | Twisted | Present | Absent | Absent | Green group | Sparse | Green | Thick | Medium | Coppery |
| **15** | Erect | Light green | Green with red streaks | Ovate | Obtuse | Rounded | Twisted | Present | Present | Absent | Green group | Sparse | Dark green | Intermediate | Medium | Coppery |
| **16** | Drooping | Whitish | Green with red streaks | Oblanceolate | Acute | Rounded | Straight | Present | Present | Absent | Green group | Sparse | Green | Thick | Dense | Red |
| **17** | Erect | Light green | Dark red | Ovate | Acute | Obtuse | Straight | Present | Present | Absent | Green group | Sparse | Light green | Thin | Sparse | Brick red |
| **18** | Drooping | Creamy | Green | Lanceolate | Obtuse | Rounded | Twisted | Present | Absent | Absent | Green group | Sparse | Green | Thin | Absent | Coppery |
| **19** | Spreading | Whitish | Green | Obovate | Obtuse | Rounded | Twisted | Present | Absent | Absent | Green group | Sparse | Dark green | Intermediate | Dense | Red |
| **20** | Spreading | Light green | Dark red | Oblong | Acute | Cordate | Twisted | Present | Absent | Absent | Green group | Sparse | Green | Thin | Absent | Red |
| **21** | Spreading | Light green | Dark red | Oblong | Obtuse | Cordate | Straight | Present | Present | Absent | Green group | Sparse | Dark green | Thick | Very dense | Red |
| **22** | Erect | Creamy | Green with red streaks | Oblanceolate | Obtuse | Obtuse | Straight | Present | Present | Absent | Green group | Sparse | Light green | Thick | Sparse | Brick red |
| **23** | Drooping | Whitish | Green with red streaks | Obovate | Rounded | Rounded | Twisted | Present | Absent | Absent | Green group | Sparse | Green | Intermediate | Dense | Brick red |
| **24** | Erect | Whitish | Green with red streaks | Oblong | Obtuse | Rounded | Straight | Present | Present | Absent | Green group | Sparse | Dark green | Intermediate | Absent | Coppery |
| **25** | Erect | Creamy | Green with red streaks | Ovate | Acute | Rounded | Twisted | Present | Absent | Absent | Green group | Sparse | Dark green | Thick | Sparse | Coppery |
| **26** | Spreading | Whitish | Green with red streaks | Obovate | Obtuse | Rounded | Twisted | Present | Absent | Absent | Green group | Sparse | Green | Thin | Absent | Red |
| **27** | Erect | Light green | Green | Oblong | Obtuse | Rounded | Twisted | Present | Absent | Absent | Green group | Sparse | Green | Intermediate | Dense | Brown |
| **28** | Spreading | Light green | Green | Obovate | Obtuse | Rounded | Straight | Present | Absent | Absent | Green group | Sparse | Light green | Thick | Absent | Coppery |
| **29** | Spreading | Creamy | Green with red streaks | Oblong | Acute | Obtuse | Straight | Present | Present | Absent | Green group | Sparse | Light green | Thick | Absent | Coppery |
| **30** | Drooping | Creamy | Green | Ovate | Rounded | Rounded | Twisted | Present | Absent | Absent | Green group | Sparse | Green | Intermediate | Sparse | Coppery |
| **31** | Spreading | Light green | Green | Obovate | Obtuse | Cordate | Straight | Present | Absent | Absent | Green group | Sparse | Dark green | Intermediate | Sparse | Coppery |
| **32** | Erect | Light green | Green with red streaks | Ovate | Obtuse | Cordate | Straight | Absent | Present | Absent | Green group | Sparse | Green | Thin | Dense | Brick red |
| **33** | Drooping | Whitish | Green | Ovate | Obtuse | Rounded | Twisted | Present | Absent | Absent | Green group | Sparse | Green | Intermediate | Dense | Brick red |
| **34** | Spreading | Creamy | Dark red | Oblong | Rounded | Rounded | Twisted | Absent | Present | Absent | Green group | Sparse | Green | Thick | Sparse | Brick red |
| **35** | Erect | Whitish | Green | Oblanceolate | Obtuse | Obtuse | Twisted | Present | Absent | Absent | Green group | Sparse | Dark green | Intermediate | Very dense | Coppery |
| **36** | Erect | Creamy | Green with red streaks | Oblong | Acute | Rounded | Twisted | Present | Present | Absent | Green group | Sparse | Dark green | Thick | Dense | Coppery |
| **37** | Spreading | Creamy | Green | Oblanceolate | Obtuse | Cordate | Twisted | Absent | Absent | Absent | Green group | Sparse | Light green | Intermediate | Sparse | Coppery |
| **38** | Drooping | Whitish | Green with red streaks | Ovate | Rounded | Rounded | Straight | Present | Absent | Absent | Green group | Sparse | Green | Thick | Dense | Coppery |
| **39** | Spreading | Light green | Green | Ovate | Rounded | Cordate | Twisted | Present | Absent | Absent | Green group | Sparse | Dark green | Thin | Very dense | Red |
| **40** | Spreading | Light green | Green with red streaks | Ovate | Obtuse | Rounded | Twisted | Absent | Absent | Absent | Green group | Sparse | Green | Thick | Sparse | Coppery |
| **41** | Spreading | Creamy | Dark red | Obovate | Obtuse | Rounded | Twisted | Present | Absent | Absent | Green group | Sparse | Dark green | Thick | Very dense | Brick red |
| **42** | Erect | Whitish | Green | Ovate | Obtuse | Rounded | Straight | Present | Absent | Absent | Green group | Sparse | Green | Intermediate | Dense | Red |
| **43** | Erect | Light green | Green with red streaks | Ovate | Obtuse | Obtuse | Twisted | Present | Absent | Absent | Green group | Sparse | Green | Intermediate | Absent | Pink |
| **44** | Spreading | Creamy | Green | Oblanceolate | Acute | Cordate | Twisted | Present | Absent | Absent | Green group | Sparse | Green | Thick | Absent | Brown |
| **45** | Spreading | Creamy | Green | Ovate | Obtuse | Rounded | Straight | Absent | Absent | Absent | Green group | Sparse | Dark green | Thin | Very dense | Brown |
| **46** | Spreading | Light green | Green with red streaks | Ovate | Rounded | Rounded | Twisted | Present | Absent | Absent | Green group | Sparse | Dark green | Intermediate | Sparse | Brown |
| **47** | Erect | Light green | Green with red streaks | Obovate | Rounded | Obtuse | Twisted | Absent | Absent | Absent | Green group | Sparse | Green | Intermediate | Sparse | Brick red |
| **48** | Spreading | Creamy | Green with red streaks | Obovate | Rounded | Cordate | Straight | Present | Absent | Absent | Green group | Sparse | Dark green | Thick | Sparse | Red |
| **49** | Erect | Light green | Green | Ovate | Obtuse | Rounded | Straight | Absent | Absent | Absent | Green group | Sparse | Light green | Thin | Dense | Brown |
| **50** | Erect | Light green | Green | Obovate | Rounded | Obtuse | Twisted | Present | Absent | Absent | Green group | Sparse | Green | Thin | Absent | Coppery |

**S. Table 2.** Variations in the fruit qualitative morphological parameters among newly developed guava hybrids and parental genotypes.

| **S. No.** | **Guava genotype** | **Fruit: shape at stalk end** | **Fruit: prominence of neck** | **Fruit: relief of surface** | **Fruit: Longitudinal ridges** | **Fruit: Longitudinal grooves** | **Fruit: puffiness** | **Fruit: peel color** | **Pulp: color** |
| --- | --- | --- | --- | --- | --- | --- | --- | --- | --- |
| **P_1_** | Allahabad Safeda | Pointed | Present | Smooth | Absent | Absent | Absent | Yellow Group (3C) | White Group (155D) |
| **P_2_** | Arka Kiran | Rounded | Absent | Rough | Present | Absent | Absent | Green Yellow Group (1C) | Red Group (39B) |
| **1** | GH20_1A | Rounded | Absent | Smooth | Absent | Absent | Absent | Yellow Group (8C) | Red Group (38B) |
| **2** | GH20_1B | Pointed | Absent | Smooth | Absent | Absent | Absent | Yellow Group (3C) | Red Group (48B) |
| **3** | GH20_1C | Rounded | Absent | Rough | Present | Absent | Absent | Green Yellow Group (1A) | Red Group (38C) |
| **4** | GH20_1D | Rounded | Absent | Smooth | Absent | Absent | Absent | Yellow Group (4C) | Red Group (48C) |
| **5** | GH20_10D | Rounded | Absent | Rough | Present | Absent | Absent | Yellow Group (2C) | Red Group(49A) |
| **6** | GH20_2B | Rounded | Absent | Smooth | Absent | Present | Absent | Yellow Orange Group (14C) | Red Group (38A) |
| **7** | GH20_2C | Necked | Present | Rough | Present | Absent | Absent | Yellow Group (2B) | Red Group (38A) |
| **8** | GH20_2E | Rounded | Absent | Smooth | Absent | Absent | Absent | Yellow Group (2C) | Red Group (38A) |
| **9** | GH20_3A | Rounded | Present | Rough | Present | Absent | Absent | Green Yellow Group (1A) | Red Group (48B) |
| **10** | GH20_3B | Rounded | Absent | Smooth | Absent | Absent | Absent | Green Yellow Group (1B) | Red Group (38B) |
| **11** | GH20_3C | Broadly Rounded | Absent | Rough | Present | Absent | Absent | Yellow Group (5C) | Red Group (38B) |
| **12** | GH20_3D | Rounded | Absent | Smooth | Absent | Absent | Absent | Green Yellow Group (1B) | Red Group (38A) |
| **13** | GH20_4B | Pointed | Present | Smooth | Absent | Absent | Absent | Green Yellow Group (1B) | Red Group (38D) |
| **14** | GH20_5A | Rounded | Absent | Smooth | Absent | Absent | Absent | Green Yellow Group (1B) | Red Group (38A) |
| **15** | GH20_5C | Pointed | Absent | Rough | Present | Absent | Absent | Green Yellow Group (1A) | Red Group (38C) |
| **16** | GH20_5D | Necked | Present | Rough | Present | Present | Absent | Green Yellow Group (1B) | Red Group (38B) |
| **17** | GH20_5E | Necked | Present | Rough | Present | Present | Absent | Yellow Group (2B) | Red Group (38C) |
| **18** | GH20_6A | Rounded | Absent | Smooth | Absent | Absent | Absent | Green Yellow Group (1C) | Red Group (38B) |
| **19** | GH20_6B | Broadly Rounded | Absent | Smooth | Absent | Absent | Absent | Yellow Orange Group (14D) | Red Group (48C) |
| **20** | GH20_6D | Rounded | Absent | Smooth | Absent | Absent | Absent | Yellow Group (13C) | Red Group (38D) |
| **21** | GH20_6E | Rounded | Absent | Smooth | Absent | Present | Absent | Yellow Group (5D) | Red Group (38D) |
| **22** | GH20_7C | Rounded | Absent | Smooth | Absent | Present | Absent | Yellow Group (12C) | Red Group (48D) |
| **23** | GH20_7E | Pointed | Present | Smooth | Absent | Present | Absent | Green Yellow Group (1C) | Red Group (36B) |
| **24** | GH20_8A | Rounded | Absent | Smooth | Absent | Absent | Absent | Yellow Group (11B) | Red Group (37A) |
| **25** | GH20_8B | Pointed | Absent | Smooth | Absent | Present | Absent | Yellow Group (3B) | Red Group (38A) |
| **26** | GH20_8C | Rounded | Absent | Rough | Present | Absent | Absent | Yellow Green Group (150A) | Red Group (38B) |
| **27** | GH20_8D | Pointed | Absent | Smooth | Absent | Present | Absent | Yellow Group (2B) | Red Group (38D) |
| **28** | GH20_8E | Rounded | Absent | Smooth | Absent | Present | Absent | Yellow Group (5C) | Red Group (48D) |
| **29** | GH20_9A | Broadly Rounded | Absent | Smooth | Absent | Absent | Absent | Yellow Group (5B) | Red Group (37C) |
| **30** | GH20_9B | Pointed | Absent | Rough | Present | Absent | Absent | Green Yellow Group (1B) | Red Group (38B) |
| **31** | GH20_10A | Pointed | Absent | Smooth | Absent | Absent | Absent | Green Yellow Group (1A) | Red Group (38C) |
| **32** | GH20_11B | Rounded | Absent | Smooth | Absent | Absent | Absent | Yellow Group (6D) | Red Group (38A) |
| **33** | GH20_11C | Pointed | Absent | Rough | Present | Present | Absent | Yellow Group (1B) | Red Group (39C) |
| **34** | GH20_11D | Rounded | Absent | Smooth | Absent | Absent | Absent | Yellow-Green Group (150A) | Red Group(49A) |
| **35** | GH20_12B | Rounded | Absent | Rough | Present | Absent | Absent | Yellow Group (2A) | Red Group(49A) |
| **36** | GH20_12E | Rounded | Absent | Smooth | Absent | Absent | Absent | Yellow Group (4C) | Red Group (38C) |
| **37** | GH20_13B | Rounded | Absent | Smooth | Absent | Absent | Absent | Yellow Group (2C) | Red Group (38C) |
| **38** | GH20_13E | Pointed | Absent | Rough | Absent | Absent | Absent | Yellow-Green Group (150A) | Red Group (38B) |
| **39** | GH20_14D | Pointed | Absent | Smooth | Absent | Absent | Absent | Yellow Group (1B) | Red Group (38B) |
| **40** | GH20_15B | Rounded | Absent | Smooth | Absent | Absent | Absent | Yellow Group (1B) | Red Group(49A) |
| **41** | GH20_15E | Rounded | Absent | Rough | Present | Absent | Absent | Yellow Group (3C) | Red Group (38B) |
| **42** | GH20_18B | Rounded | Absent | Smooth | Absent | Absent | Present | Yellow Group (2C) | Red Group (38C) |
| **43** | GH20_16D | Rounded | Absent | Smooth | Absent | Absent | Absent | Yellow-Green Group (150B) | Red Group (38A) |
| **44** | GH20_17B | Rounded | Absent | Smooth | Absent | Absent | Present | Yellow Group (2B) | Red Group (38A) |
| **45** | GH20_17E | Pointed | Present | Smooth | Absent | Absent | Absent | Yellow Group (2B) | Red Group (38A) |
| **46** | GH20_5B | Rounded | Absent | Rough | Present | Present | Absent | Yellow Group (3B) | White Group (NN155A) |
| **47** | GH20_11A | Broadly Rounded | Absent | Rough | Present | Absent | Absent | Green Yellow Group (1C) | White Group (NN155B) |
| **48** | GH20_12C | Rounded | Absent | Rough | Present | Absent | Absent | Green Yellow Group (1A) | White Group (NN155B) |
| **49** | GH20_18A | Broadly Rounded | Absent | Rough | Present | Present | Absent | Yellow Group (3B) | White Group (NN155D) |
| **50** | GH20_20B | Rounded | Absent | Rough | Present | Absent | Absent | Yellow Group (2A) | White Group (NN155A) |

**S. Table 3.** Principal component analysis for MGIDI.

| **S. No** | **PC** | **Eigenvalues** | **Variance (%)** | **Cum. variance (%)** |
| --- | --- | --- | --- | --- |
| 1 | PC1 | 2.38 | 19.80 | 19.80 |
| 2 | PC2 | 2.23 | 18.61 | 38.41 |
| 3 | PC3 | 2.04 | 16.99 | 55.40 |
| 4 | PC4 | 1.47 | 12.25 | 67.65 |
| 5 | PC5 | 1.17 | 9.75 | 77.40 |
| 6 | PC6 | 0.83 | 6.93 | 84.33 |
| 7 | PC7 | 0.66 | 5.47 | 89.80 |
| 8 | PC8 | 0.54 | 4.52 | 94.32 |
| 9 | PC9 | 0.29 | 2.41 | 96.73 |
| 10 | PC10 | 0.20 | 1.67 | 98.40 |
| 11 | PC11 | 0.13 | 1.12 | 99.52 |
| 12 | PC12 | 0.06 | 0.48 | 100.00 |

**S. Table 4.** Eigen values, Variance (%) and cumulative variance (%) of the five principal component axes from the PCA of morpho-biochemical parameters in the studied hybrids and their parents.

| **S. No** | **Parameters** | **Dim.1** | **Dim.2** | **Dim.3** | **Dim.4** | **Dim.5** |
| --- | --- | --- | --- | --- | --- | --- |
| 1 | Leaf length | 0.52 | -0.4 | 0.38 | -0.09 | 0.13 |
| 2 | Leaf width | 0.34 | -0.46 | 0.06 | -0.04 | 0.13 |
| 3 | Leaf length/width | 0.2 | 0.1 | 0.39 | -0.09 | -0.01 |
| 4 | Petiole length | 0.38 | -0.3 | -0.15 | 0.36 | -0.07 |
| 5 | Fruit length | **0.73**** | -0.18 | -0.03 | -0.43 | 0.26 |
| 6 | Fruit width | **0.84**** | -0.34 | -0.01 | -0.05 | -0.11 |
| 7 | Fruit length/width | -0.03 | 0.15 | -0.02 | -0.5 | 0.5 |
| 8 | Fruit weight | **0.91**** | -0.23 | -0.06 | -0.11 | 0.08 |
| 9 | Fruit thickness of outer pulp | **0.71**** | -0.25 | 0.04 | -0.17 | 0.08 |
| 10 | Fruit diameter of calyx | 0.35 | -0.16 | 0.2 | 0.18 | -0.33 |
| 11 | TCD pulp | -0.04 | -0.31 | -**0.58**** | 0.45 | -0.04 |
| 12 | TCD peel | 0.14 | -0.21 | -0.33 | 0.42 | -0.38 |
| 13 | TSS | 0.57 | **0.67**** | -0.2 | 0.09 | 0.24 |
| 14 | TA | -0.16 | -0.37 | -0.02 | 0.46 | **0.66**** |
| 15 | TSS/TA | 0.25 | 0.44 | -0.02 | -0.29 | -0.7 |
| 16 | Ascorbic acid | 0.48 | -0.1 | 0.31 | 0.41 | -0.05 |
| 17 | Lycopene | 0.28 | -0.27 | 0.47 | 0.02 | -0.23 |
| 18 | Total Carotenoids | -0.15 | 0.42 | 0.6 | -0.23 | 0.09 |
| 19 | Total phenol content | 0.07 | 0.33 | 0.47 | 0.32 | -0.05 |
| 20 | Total flavonoids | 0.17 | 0.14 | 0.21 | 0.09 | -0.24 |
| 21 | DPPH | 0.01 | 0.13 | 0.47 | 0.53 | 0.09 |
| 22 | FRAP | 0.03 | 0.36 | 0.46 | 0.42 | 0.26 |
| 23 | Total sugars | 0.54 | **0.72**** | -0.3 | 0.17 | 0.11 |
| 24 | Reducing Sugar | 0.47 | **0.63**** | -0.11 | 0.1 | 0.17 |
| 25 | Non-reducing sugar | 0.42 | 0.55 | -0.4 | 0.17 | 0.01 |
|  | **Eigenvalue** | **4.73** | **3.45** | **2.49** | **2.21** | **1.85** |
|  | **% of Variance** | **18.93** | **13.81** | **9.96** | **8.85** | **7.42** |
|  | **Cumulative %** | **18.93** | **32.74** | **42.70** | **51.57** | **58.99** |

** Eigenvalues are significant ≥ 0.58

**S. Table 5.** Mean weekly weather data during July 2024 to May 2025.

| **Week** **No.** | **T_max_ (°C)** | **T_min_ (°C)** | **Rainfall (mm)** | **RH mean (%)** |
| --- | --- | --- | --- | --- |
| **1** | 34.43 | 27.67 | 3.26 | 86.14 |
| **2** | 35.13 | 27.60 | 3.59 | 85.86 |
| **3** | 36.37 | 28.73 | 4.14 | 83.43 |
| **4** | 34.20 | 27.57 | 2.89 | 91.86 |
| **5** | 35.56 | 28.23 | 3.83 | 85.29 |
| **6** | 33.94 | 26.17 | 3.26 | 88.71 |
| **7** | 32.69 | 26.70 | 2.30 | 90.00 |
| **8** | 34.53 | 27.53 | 2.88 | 87.50 |
| **9** | 34.07 | 25.83 | 2.86 | 91.29 |
| **10** | 33.56 | 25.63 | 2.56 | 90.71 |
| **11** | 31.09 | 23.96 | 1.80 | 93.14 |
| **12** | 32.81 | 24.89 | 2.46 | 88.57 |
| **13** | 35.47 | 26.16 | 3.09 | 84.43 |
| **14** | 35.46 | 24.80 | 3.63 | 80.43 |
| **15** | 34.50 | 21.01 | 4.07 | 81.29 |
| **16** | 35.21 | 18.27 | 3.77 | 84.57 |
| **17** | 33.63 | 18.99 | 3.53 | 87.00 |
| **18** | 33.71 | 18.23 | 3.33 | 83.29 |
| **19** | 31.83 | 16.87 | 2.89 | 91.57 |
| **20** | 30.40 | 15.47 | 2.67 | 88.14 |
| **21** | 26.19 | 10.63 | 2.06 | 88.14 |
| **22** | 26.29 | 8.99 | 2.51 | 89.29 |
| **23** | 25.67 | 7.94 | 2.53 | 82.43 |
| **24** | 22.17 | 5.23 | 2.29 | 77.14 |
| **25** | 22.09 | 4.27 | 1.89 | 85.29 |
| **26** | 19.21 | 10.57 | 1.19 | 93.71 |
| **27** | 16.90 | 7.00 | 0.40 | 93.00 |
| **28** | 17.50 | 6.90 | 0.40 | 92.10 |
| **29** | 17.30 | 8.60 | 1.20 | 92.40 |
| **30** | 23.60 | 8.70 | 0.00 | 87.90 |
| **31** | 23.70 | 7.40 | 0.00 | 90.00 |
| **32** | 23.50 | 10.30 | 0.00 | 84.10 |
| **33** | 26.40 | 10.30 | 0.00 | 77.30 |
| **34** | 27.20 | 11.70 | 0.10 | 86.40 |
| **35** | 27.30 | 14.80 | 0.30 | 82.70 |
| **36** | 28.90 | 10.80 | 0.00 | 75.90 |
| **37** | 33.30 | 16.90 | 0.10 | 84.00 |
| **38** | 31.50 | 14.40 | 0.00 | 80.40 |
| **39** | 35.30 | 15.80 | 0.00 | 69.70 |
| **40** | 36.20 | 16.00 | 0.00 | 69.30 |
| **41** | 38.30 | 22.00 | 0.00 | 70.60 |
| **42** | 38.10 | 23.60 | 0.00 | 68.00 |
| **43** | 40.90 | 20.80 | 0.00 | 56.60 |
| **44** | 36.50 | 23.00 | 7.40 | 71.70 |
