# Supplementary material for: Multivariate assessment of newly developed guava (Psidium guajava L.) hybrids for tree and fruit quality traits
Source: Sci Rep. 2026 Apr 4;16:16246. doi: 10.1038/s41598-026-45320-8 (PMC13201807; doi:10.1038/s41598-026-45320-8)
Supplement: Supplementary file 2 — Supplementary Material 2 [file 41598_2026_45320_MOESM2_ESM.docx]

**
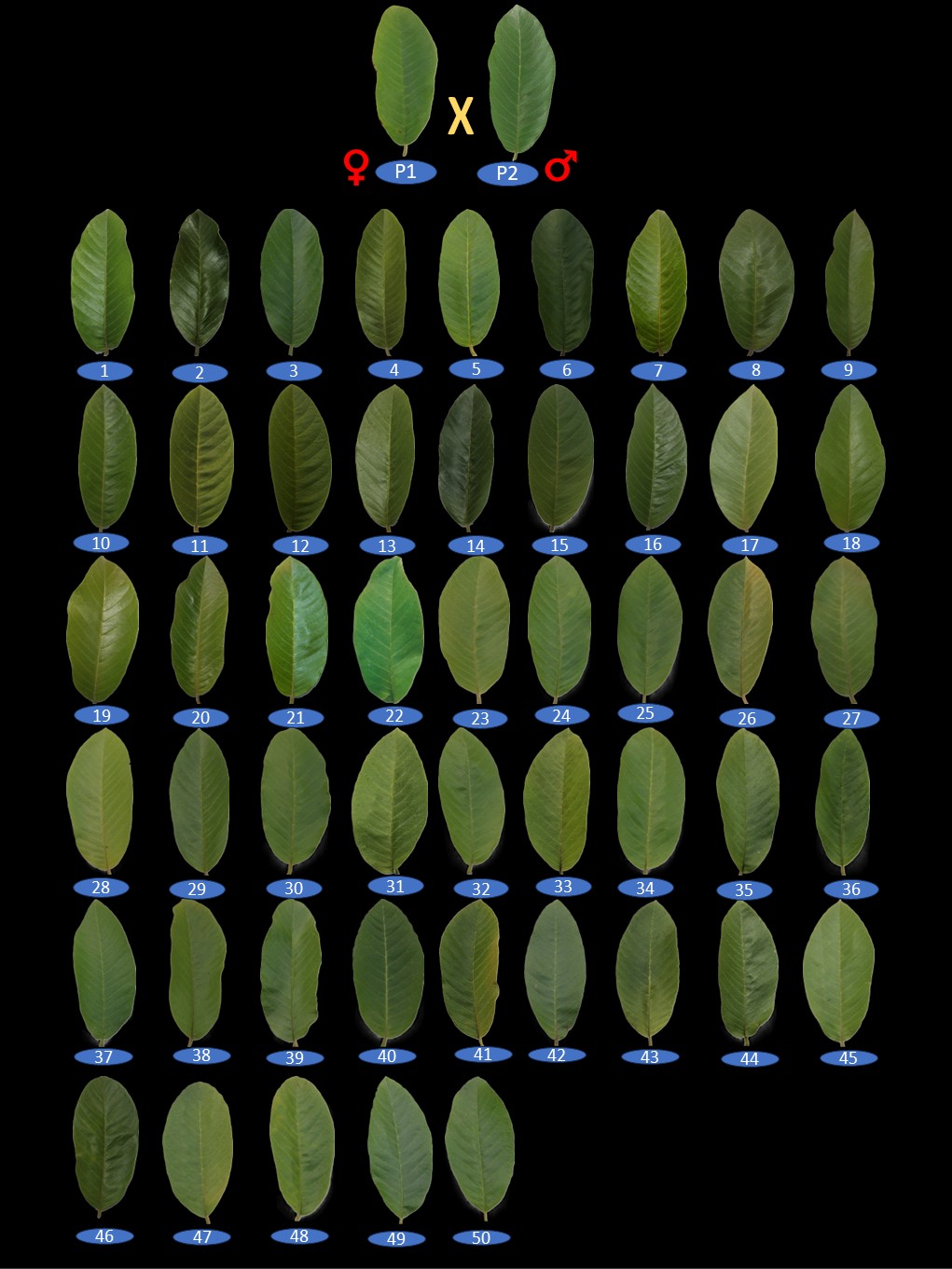
**

**S. Fig. 1 Variations in leaf morphological parameters among newly develop guava hybrid progenies and their parents.**

(**Where**; **P_1_**-Allahabad Safeda, **P_2_**_-_Arka Kiran, **1.** GH20_1A, **2**. GH20_1B, **3.** GH20_1C, **4.** GH20_1D, **5.** GH20_10D, **6.** GH20_2B, **7.** GH20_2C, **8.** GH20_2E, **9.** GH20_3A, **10.** GH20_3B, **11.** GH20_3C, **12.** GH20_3D, **13.** GH20_4B, **14.** GH20_5A, **15.** GH20_5C, **16.** GH20_5D, **17.** GH20_5E, **18.** GH20_6A, **19.** GH20_6B, **20.** GH20_6D, **21.** GH20_6E, **22.** GH20_7C, **23.** GH20_7E, **24.** GH20_8A, **25.** GH20_8B, **26.** GH20_8C, **27.** GH20_8D, **28.** GH20_8E, **29.** GH20_9A, **30.** GH20_9B, **31.** GH20_10A, **32.** GH20_11B, **33.** GH20_11C, **34.** GH20_11D, **35.** GH20_12B, **36.** GH20_12E, **37.** GH20_13B, **38.** GH20_13E, **39.** GH20_14D, **40.** GH20_15B, **41.** GH20_15E , **42.** GH20_18B, **43.** GH20_16D,**44.** GH20_17B, **45.** GH20_17E, **46.** GH20_5B, **47.** GH20_11A, **48**. GH20_12C, **49.** GH20_18A, **50.** GH20_20B.)


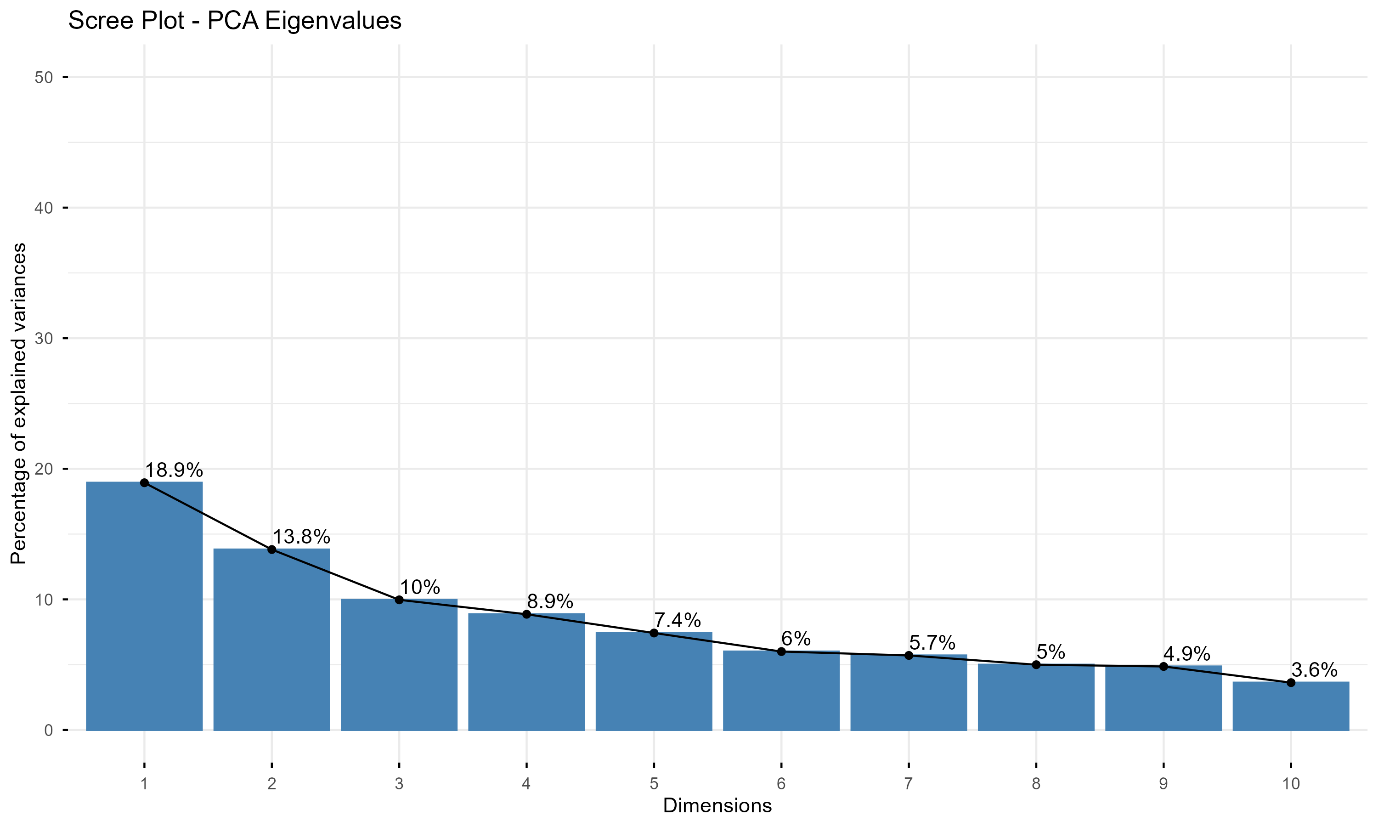


**S. Fig. 6** **Scree plot, showing variance explained by principal components derived from morpho-biochemical parameters among the hybrid progenies and their parents.**
